# Climatic preferences of major plant clades define the functional attributes of African savanna types

**DOI:** 10.1038/s44185-026-00138-5

**Published:** 2026-05-23

**Authors:** Vitus Pickelmann, Steven I. Higgins

**Affiliations:** Plant Ecology, University of Bayreuth, Bayreuth, Germany

**Keywords:** Ecology, Ecology, Evolution, Plant sciences

## Abstract

Forecasting how the Earth system will respond to global change includes simplifying the functional diversity induced by thousands of plant species into tractable units. The dominant strategy has been to aggregate species into plant functional types (PFTs), assuming that selection leads to unrelated species converging to similar ecological roles. An alternative would be to harness niche conservatism, the tendency of lineages to retain ancestral ecological traits, and assume that phylogenetically related species tend to have similar ecological roles. Using African savannas as case study, we use phylogenetically defined groups to identify phytoclimes: climate-defined regions that support distinct sets of plant types. We found that taxonomically-based phytoclimes aligned with a respected expert map of Africa. Moreover, this scheme could be reconciled with existing conceptual models used to describe the functional diversity of savannas. This alignment of phylogenetic and functional interpretations suggests that African savannas are dominated by a limited set of pre-adapted taxa, rather than arising through widespread convergence. Our findings suggest that phylogenetic information can provide a parsimonious, functional basis for representing ecosystem diversity in global change models. Harnessing this alignment between phylogenetic and functional groupings offers a promising route for improving the predictive ability of Earth system models.

## Introduction

Predicting the functional attributes and state of ecosystems is a foundational and pressing aim in ecology. To address this challenge while accommodating the complexity of ecosystems early dynamic global vegetation models^1^ focused on seed plants and abstracted the circa 350,000 species of seed plants into a small (<50) number of plant functional types (PFTs)^2^. These functional types represented hypotheses of broad categories of response types to abiotic forcing and biotic interactions. The appeal of PFTs lies in their promise that a mechanistic understanding of growth form and leaf economic traits might provide an appropriate foundation for simulating the essential dynamics of ecosystems without needing to parameterize species-level variation.

The PFT approach still underpins the representation of the land surface in current generation Earth system models^3^ and usage of the biome concept in ecology^4,5^. Despite its widespread adoption, the PFT paradigm has delivered only moderate predictive success ^6^. Understanding this lack of success involves reexamining two assumptions that underpin the use of PFTs in Earth system models. First, it is assumed that evolutionary convergence is sufficiently widespread that unrelated species in similar environments evolve similar PFT syndromes^4^. Second, it assumes that these PFTS can be meaningfully defined and operationalised for use in models. Neither assumption holds universally.

An alternative approach is to acknowledge that niche conservatism, defined as the tendency of species to retain ancestral ecological traits^7^, may play a dominant role in structuring plant communities. If niche conservatism were dominant, communities would be assembled primarily from pre-adapted lineages, leading to functional similarity driven by shared evolutionary history. In contrast, if niche convergence were dominant, communities would be composed of species that have independently evolved similar functional traits in situ, resulting in functional similarity that is decoupled from phylogeny. Implicit in both cases, is that community assembly is constrained by limiting similarity, which precludes the coexistence of functionally identical species locally. In reality, the traits of species in local communities reflect the combined influence of niche conservatism and niche convergence as community assembly is highly complex^8^. Although this tension between niche conservatism and niche convergence is well studied in phylogenetic community ecology ^9,10^, the consequences for Earth system modelling have not been thoroughly explored. If niche conservatism were a dominant force, molecular phylogenies could be used to identify functional groups, side-stepping the difficulties associated with defining PFTs using traits.

Although Earth system models have almost exclusively represented vegetation using PFTs under the evolutionary convergence assumption, the fields of vegetation science, conservation biology and biogeography acknowledge niche conservatism by using floristic criteria to make ecosystem maps. These maps, often termed regionalisations^11^, explicitly acknowledge the importance of niche conservatism, dispersal limitations and other historical contingencies. For example, Daru et al. showed the potential of using phylogenetic information to cluster functionally relevant units in geographic space in a previous study in Africa^12^. However, regionalisation studies are usually directed toward identifying centres of endemism, describing evolutionary radiations, and setting conservation priorities, and thus remain largely disconnected from the predictive agendas of functional ecology, global change ecology and Earth system modelling.

In this study, we pose the hypothesis that a priori defined phylogenetic groups can provide more information for the classification and mapping of the distribution of ecosystems than PFT-based methods. Although the use of phylogenetic relatedness as a proxy for functional relatedness comes with caveats, in systems where a few taxa dominate, phylogenetic relatedness may associate with functional attributes^13^. To explore this idea we use the concept of a phytoclime, a region where climate favours the growth of similar combinations of plant types^14^, to examine whether phylogenetically defined plant types can provide useful classifications of ecosystems. We use African savannas as a tractable case study to illustrate the promise of this idea. Informed by the literature on savanna types^15–19^, we use (paraphyletic) indicator taxonomic groups, that is members of selected families, subfamilies and genera.

### The case study

African savannas are defined as ecosystems with a grassy understorey of C4 grass species and a sparse overstorey of shade intolerant trees^20^. Ecosystems fitting this definition take on many faces: the grass-rich Serengeti, the southern African Miombo woodlands, the Guinean savanna belt and the central Kalahari all fit this savanna definition. Linked to this coherent definition is a solid theoretical understanding of how savannas work^21^; however, there is less consensus on how to classify the functional differences between savannas, manifested by the variety of schemes that have been proposed ^15,17,19,22–28^.

Classifications of African savannas can be split into floristic^15–17,19^ and functional^23,25,28,29^, but closer inspection reveals that the floristic and functional attributes are often confounded. For example, the sweet- and sourveld scheme, which is a functional concept motivated by agronomic aims^23^, uses taxonomically and PFT-based groups. Sweetveld entails fast-open nutrient cycling, high palatability, low plant biomass and is typically associated with a balanced grass taxonomic composition and the taxon *Vachellia* (and the paraphyletic *Senegalia*) dominating the tree layer. Sourveld, in contrast entails slow-closed nutrient cycling, low palatability, high plant biomass, where the typical savanna grass taxa Aristidoideae and Chloridoideae are absent and Combretaceae and Detarioideae dominate the tree layer^17,25,28^. The distribution of sweetveld or sourveld has been linked to external drivers such as climate and soil fertility^25,28,30–33^. For example, sweetveld is associated with dry climates and fertile soils, floristically, the grass layer is dominated by Chloridoids whose usage of the NAD-ME C4 pathway has been linked to high nutrient requirements and a tolerance of aridity^16,17,28,34^. Typical sweetveld tree taxa often form nitrogen-fixing relationships with rhizobial bacteria (e.g. *Acacia* s.l.: *Vachellia* and *Senegalia*) often interpreted as an adaptation to soils high in base minerals but low in nitrogen^35,36^. By contrast, sourveld is more common in high rainfall, nutrient poor environments and is characterised by Panicoideae, which predominantly use the NADP-ME C4 pathway. High rainfall is typically associated with leached soils, which selects for tree taxa that enter into ectomychorrhizal relationships (Detarioideae), or in low base mineral situations, with arbuscular mycorrhiza (Combretaceae)^16,34,36^. Sweetveld - sourveld is not only linked to climate and soils, but also to differences in the internal dynamics associated with fire and herbivory^25,37,38^. For example, sweetveld has been interpreted as meso-herbivore controlled (eutrophic savanna) and sourveld as fire-controlled (dystrophic savanna)^25,37^. Extremely arid environments tend to fall outside the sweetveld-sourveld continuum: here the grass layer is characterized by Aristidoideae, which rely largely on the PCK C4 pathway, which is regarded as an adaptation to extremely arid and nutrient-poor habitats. Characteristic trees in such arid conditions are members of the genus *Commiphora*.

The literature reviewed in the previous paragraph reveals a confounding of functional and phylogenetic attributes of sweet- and sourveld savannas, which suggest that these ecosystems are structured by ecological trait filtering rather than recent convergent evolutionary adaptation. In particular, the dominance of a small number of taxonomic groups with distinctive functional profiles implies that these taxa were pre-adapted to the savanna environment, which only emerged in the late Miocene (11 - 5 million years ago)^39^, too recent for maladapted taxa to evolve equivalent adaptations or to overcome the priority advantage of the pre-adapted groups^40,41^. Thus, niche conservatism of the indicator savanna taxa may be the dominant structuring factor in African savannas, which may make phylogenetic information a better proxy for function than traits or life forms. This then leads us to hypothesize that savanna types in Africa can accurately be classified by defining the climatic preferences of the dominant indicator savanna taxa.

To achieve this classification we focussed on the seven taxonomic groups identified in the previous paragraphs, namely *Vachellia*, *Commiphora*, Combretaceae, Detarioideae, Panicoideae, Chloridoideae, Aristidoideae. For these groups we compiled a data set of occurrence data for African savanna regions using a combination of presence-only and presence-absence data sources. We then used an existing workflow^42^ to describe the climatic preferences of each of these taxonomic groups. This phytoclimatic workflow involved fitting a species distribution model^43,44^ to the occurrence data for 1255 species-level occurrence data sets, using the average modelled suitability of the species in each of the seven taxonomic groups to generate taxonomic group suitability surfaces, and finally clustering sites with similar values across the seven surfaces to generate ecosystem classes. We refer to these classes as phytoclimes^14^ to distinguish the resulting map from biome maps or floristic regionalisations. The phytoclime map for Africa based on this classification was formally compared to White’s vegetation map for Africa^19^. In post-hoc analyses we used recursive partitioning to elucidate which climatic and phytoclimatic variables define the savanna types. We further assessed the extent to which the taxon-based phytoclime map can predict the functional properties of different savanna types.

## Results

### Environmental suitability mapping

**Fig. 1:**
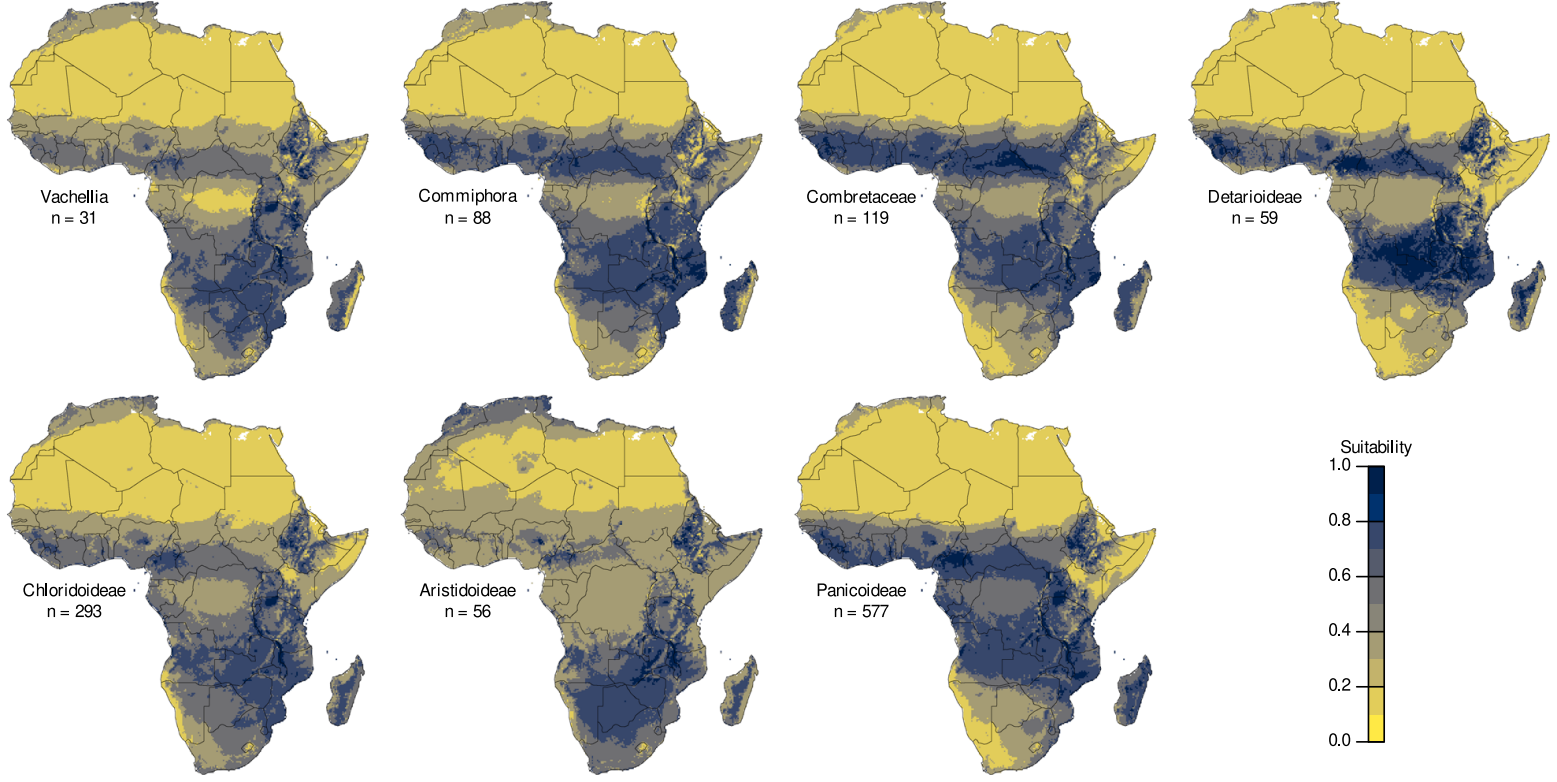
Geographic distribution of climatic suitability for seven dominant African savanna plant taxonomic groups. Suitability is calculated as the mean suitability of species belonging to each taxonomic group as estimated by a plant growth model. A total of 1248 species models were used, *n* indicates the number of species models underlying each taxonomic group’s climatic suitability surface.

Plant species distribution models were produced for 1255 tree and grass species from seven defining savanna taxonomic groups (Supplementary Table S1). The majority (99.6%) of models displayed acceptable predictive accuracy (AUC > = 0.75). The suitability surfaces generated by stacking and averaging the species distribution model suitability projections by taxonomic group displayed high suitability for all seven groups in the greater savanna region of Africa (Fig. 1). This showed that our species and taxa selections yielded models that represent plausible hypotheses of how climatic factors constrain the potential distribution of major savanna tree and grass taxonomic groups.

### Savanna classification

**Fig. 2:**
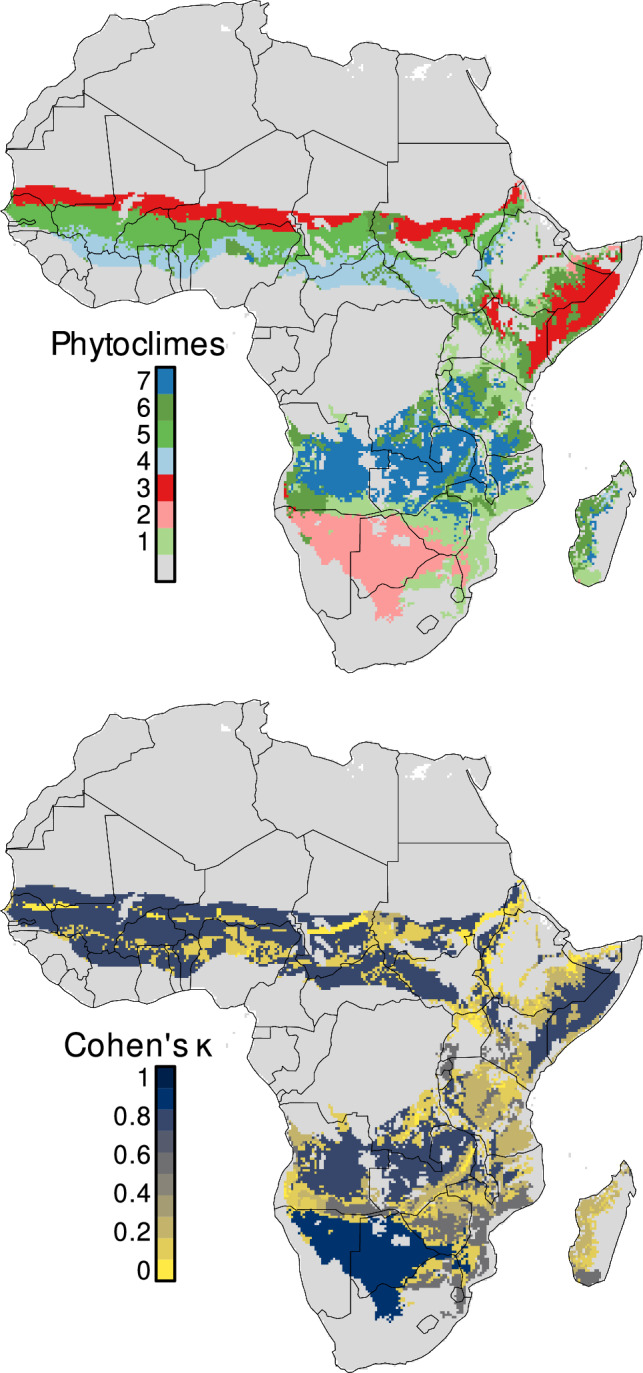
African savanna phytoclimes defined using an unsupervised classification of the taxonomic group suitability surfaces shown in Fig. 1 and the agreement between the phytoclime map and White’s vegetation map of Africa (Supplementary Fig. S1) estimated using Cohen’s *κ.* Existing conventions^86^ interpret *κ* ≤0.40 as poor agreement, 0.40 < *κ* < 0.75 as good agreement and *κ* ≥0.75 as very good agreement. Grey areas on both maps indicate non-savanna areas. Supplementary Fig. S2 shows the *κ* statistics when contrasting the White map to a published growth - form based phytoclime map^42^. For maps with different number of phytoclimes (2–10) see Supplementary Fig. S8.

**Fig. 3:**
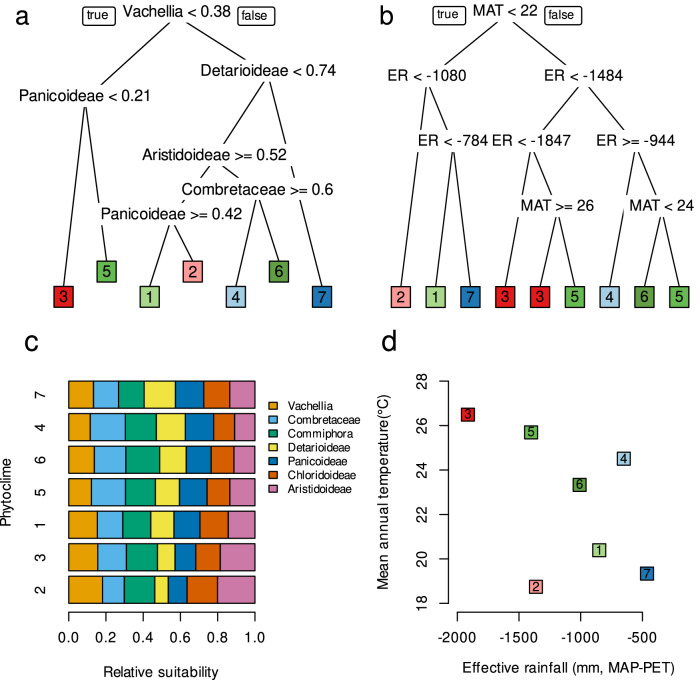
Decision trees based on recursive partitioning revealing how (**a**) plant taxonomic group and (**b**) climatic variables determine phytoclime type. MAT indicates mean annual temperature (^∘^C) and ER indicates effective rainfall (mm, ER = mean annual precipitation - Penman potential evapotranspiration). Displayed below are (**c**) the distribution of relative taxonomic group suitability in each phytoclime and (**d**) the mean position of each phytoclime in a mean annual temperature and effective rainfall biplot.

The unsupervised classification of the seven suitability surfaces yielded a classification with seven phytoclimes (Fig. 2). A post-hoc recursive partitioning was used to interpret the classes (Fig. 3). The Miombo phytoclimes 4 and 7 were defined by high Detarioideae or Combretaceae suitability, while the arid sweetveld phytoclimes 2 and 3 were connected to a low suitability for Panicoideae (Fig. 3). Regionally, the west African phytoclimes 3 and 5 could be split from the rest according to their low suitability for *Vachellia* (Fig. 3). Phytoclime 6 was somewhat taxonomically undifferentiated with its most distinctive trait being moderate *Vachellia* suitability and high suitability for *Commiphora* (Fig. 3).

Post-hoc recursive partitioning of the phytoclime classes using effective rainfall and mean annual air temperature revealed that climate could explain a substantial portion of the phytoclime classification (cross-validated error rate of 0.34 for seven classes, Fig. 3). The major division was between warm and cold phytoclimes (west African and southern-east African), while the remaining divisions were primarily related to rainfall (Fig. 3). Phytoclimes 2 and 3 were associated with very dry-cold and very dry-very hot climates, respectively (Fig. 3). Phytoclime 4 and 7 were associated with high rainfall irrespective of air temperature (Fig. 3).

### Formal map comparison

To formally compare the maps, we used an algorithm that harmonizes the number of classes on the maps and then calculates Cohen’s kappa (*κ*) for each pairing of classes^45^. Overall, 64.2% of pixels in the phytoclime map agreed better than poor and 54.2% displayed good agreement with the White map (0.75 > *κ* > 0.4; Fig. 2). About 10% of the pixels agreed very well (9.9%; *κ* ≥0.75) with White’s map (Fig. 2). Phytoclime 6 was the only one with low agreement (Fig. 2). In contrast, a growth-form based phytoclime map of Africa^42^ generated consistently lower *κ* statistics; in this case 0% of pixels displayed very good agreement, while 22.6% showed good agreement (Supplementary Fig. S2).

### Functional analysis of the classes

**Fig. 4:**
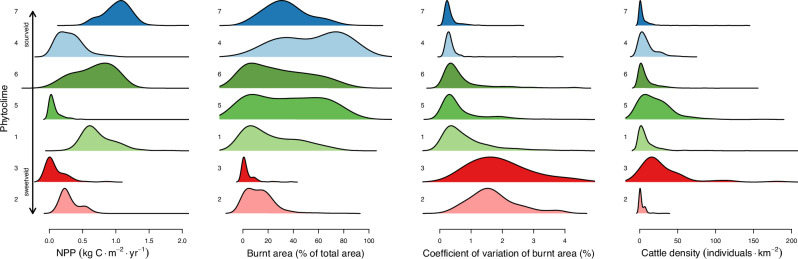
Kernel density estimates of functional attributes of each phytoclime. The phytoclimes are arranged on a sweetveld to sourveld gradient. Supplementary Fig. S3 shows an analogous plot using a published growth - form based phytoclime map^42^.

The functional value of the phytoclime scheme is further evidenced by differences in their functional attributes (Fig. 4). In particular, the west African savanna phytoclimes (3, 4 and 5) had a lower net primary productivity (NPP) than the other phytoclimes (1, 2, 6 and 7; Fig. 4) and there was a gradual trend towards higher NPP from sweet to sour phytoclimes (Fig. 4). Regarding fire activity, the sweet phytoclimes (2 and 3) had a low burnt area and a high coefficient of variation of burnt area, indicating infrequent fire return intervals (Fig. 4). The phytoclimes intermediate on the sweet- and sourveld gradient showed lower fire activity than the sour phytoclimes (4 and 7), but higher fire activity than the sweet phytoclimes (2 and 3) (Fig. 4). The phytoclimes also differed in the density of the major livestock species (cattle) they supported; however, there was no simple trend of sweetveld phytoclimes supporting higher cattle densities than sourveld phytoclimes (Fig. 4).

A fundamental motivation for classifying ecosystems is to use the classifications to predict ecosystem properties and we therefore tested whether the taxa-based classification could explain more variance in the ecosystem properties shown in Fig. 4 than an existing growth form-based classification^42^. This comparison indicated that the predictive performance of the taxa-based classification was superior across all four ecosystem properties investigated. This pattern was evident using both the WAIC and LOO criteria and the differences detected were well above thresholds typically considered decisive (Supplementary Tables S2, S3). Gelman’s R2 measure for absolute goodness-of-fit indicated that our model was able to explain a larger portion of the observed variance (Supplementary Table S4). Further, in a comparison with the floristic map of White^19^, both models performed equally well in describing variance in Net Primary Production and cattle density, however the White model described variance in both burnt area and the coefficient of variation of burnt area better than the taxa-based classification (Supplementary Tables S5, S6). The model using White’s map also improves the variance explained assessed with Gelman’s R2 (Supplementary Table S4). Here, White’s map with 26 classes is much more flexible than our map with seven classes, which could explain the slightly improved goodness-of-fit.

## Discussion

Our phytoclime mapping of African savannas based on a set of a priori identified savanna plant taxonomic groups showed strong agreement with established vegetation mappings^19,46,47^. It also aligns with a recent study that used tree composition data to show that the composition of African savannas are strongly structured by phylogeny^48^. This suggests that phylogenetically based groups are a viable alternative to plant functional trait-based groups for ecosystem classification. This study used dominant indicator taxa as a simplified proxy for phylogenetic relatedness to illustrate the promise of this approach. Furthermore, recursive partitioning elucidated that the climatic variables, effective rainfall and air temperature, were sufficient to explain the majority of the variation in the phytoclime classification. Our findings are supported by previous work^17,28,48^ that emphasized climatic factors as determinants of savanna types. Further, the value of our map is that it is entirely based on species distribution data and climatic forcing, meaning that the models can be used to project shifts in these phytoclimes and their associated functional properties with changing climates.

The suitability surfaces that underlie the phytoclime map revealed that of the sourveld taxonomic groups (Combretaceae and Detarioideae), Combretaceae had a higher suitability in the Kalahari, and a much higher suitability in the South African lowveld and the non-Miombo savannas of east Africa whereas the Detarioideae suitability was highest in the core Miombo regions, which is in agreement with expectations from the literature^18,19,23,47–51^. *Commiphora* suitability was high in the Sahel zone and horn of Africa, matching descriptions in previous work^17,19,47,48^. In east Africa, *Vachellia* suitability was highest in areas where on volcanic geology from the great African rift system (cf. ref. ^25^), matching^32^.

For the grasses, the suitability surfaces for Panicoideae, Aristidoideae and Chloridoideae aligned with a bioclimatic scheme proposed by Johnson and Tothill^16^. Our profiles also agreed with empirical climate-abundance relationships revealed by Forrestel et al.^52^ for South Africa. Aristidoideae and Chloridoideae displayed lower suitability in west Africa than southern-east Africa, which is consistent with Rattray’s African grass cover maps^53^.

Our phytoclime mapping showed substantial agreement with the established UNESCO vegetation map of Africa by White^19^, where 54.2% of pixels displayed good agreement with the White map (0.75 > *κ* > 0.4). In comparison to a purely functionally based mapping, our phylogenetically based approach showed better agreement with the White map when assessed using *κ* statistics. This suggested that the climatic niches of the dominant indicator taxa better explain the variation of savanna types than a growth form based approach^42^. It also added to the discussion that climate is likely a determinant of savanna distribution^25,32,36,46,54^. To put the agreement between the phytoclime map and the White map into context, it is illustrative to benchmark our *κ* statistics against those reported in a comparison of global biome maps^45^. When comparing global maps one might expect high consensus, since there is, in general, agreement on what a tropical forest, tundra, desert or temperate forest is. Nonetheless, this benchmark study^45^ reported very good agreement (*κ* ≥0.75) in 0% of the pixels and good agreement (0.40 < *κ* < 0.75) in only 19.3% of the map pixels.

The idea that soil nutrient status directly determines savanna types, or indirectly through its influence on herbivory^25,28,30,32,34,36,46,51,54^ or on fire^24,37,54,55^, has been widely discussed. Indeed, the importance of fire and herbivores as determinants of savanna types is now embedded in the functionally based IUCN ecosystem typology^56^ which recognizes trophic (herbivore controlled) and pyric (fire controlled) types. In this typology fire dominates in high rainfall, low nutrient, high grass biomass savannas, while herbivore impacts dominate in higher nutrient systems where canopy-openness limits predator success^25,37^. We propose that we can reconcile this apparent contradiction between climatic, consumer (fire, herbivory) and edaphic (soil fertility) determinants by accepting that each of this studies climatically determined savanna types (savanna phytoclimes) support distinct ecologies, each characterised by distinct pathways of biomass production and consumption. Acocks’s^23^ agronomically motivated sweet- and sourveld provides a useful framework for this reconciliation allowing us to arrange the seven African savanna phytoclimes along a sweetveld-sourveld gradient (Fig. 3). The Miombo-adjacent phytoclimes 4 and 7 can be interpreted as sourveld, phytoclimes 1, 5 and 6 as mixed veld, while phytoclimes 2 and 3 can be interpreted as sweetveld. Along this functional gradient, relative *Vachellia*, Chloridoideae and Aristidoideae suitability increased towards the sweet end, while relative Detarioideae and Panicoideae suitability increased towards the sour end.

Despite the phytoclime map being able to reproduce important distinctions apparent in the White^19^ map, some deficiencies were apparent. First, the mopaneveld category on the White map fell into two phytoclimes, causing a low agreement statistic in one of the two. This disagreement is because phytoclimes are climatic units (zonobiomes to use Walter’s terminology^57^), whereas White’s vegetation types used biogeographic and floristic classification criteria. Mopaneveld is in Walter’s terminology azonal, covering an intersection between alluvial soils and frost free climates^19,58^. Second, in east Africa White’s *Acacia-Commiphora* bushveld^19^ did not align with our Phytoclimes. White noted that this formation included substantial functional, taxonomic and environmental variation in addition to azonal vegetation caused by land-use^19^. White assigned southern African savannas to Miombo-, Mopane-, or “all other savannas”^19^ suggesting units that disagree with the phytoclime map. Other authors suggest that White’s “all other savannas” category could be split into Combretaceae dominated and *Acacia* s.l. dominated savanna^17,18,34^. The disagreement in east and southern Africa may be related to the complexity, diversity and lack of clear indicator taxonomic groups for trees and grasses in these regions^47,48^. East Africa, for instance, seems to be differentiated from other savannas by their abundance of Euphorbiaceae succulents and Rutaceae and Primulaceae shrubs^48^, which has not been captured by the historical classifications motivated by tree- and grass-differences in savannas^17,19,50^.

The taxa-based phytoclime classes developed here were able to partition significant components of the variance in the functional attributes of the study area. The partitioning of variance in fire activity with phytoclimes parallels distinctions made in a previous study^24^, which classified large parts of phytoclime 4 and 7 into a “frequent-cool-small” fire activity class, while the other phytoclimes generally fell into the “rare" fire activity class. The picture was more complex when predicting supported herbivore biomass, which we attempted by using cattle density data provided by the FAO^59^. The caveat here is that cattle density is not a perfect proxy of the forage value of an ecosystem, since herbivores with high body mass can tolerate lower quality forage^25,34^. Also, supplemental feeding can lead to higher cattle numbers than what would be supported normally. Wild herbivore data products suggest that our sourveld phytoclimes (phytoclimes 4 and 7) were associated with a large bodied (bulk-feeder) herbivore community and our sweetveld phytoclimes (phytoclimes 2 and 3) were associated with an arid gazelle herbivore community^26^. However, this alignment is circular since these wild herbivore data products^26^ used both rainfall and White’s vegetation types to infer herbivore biomass.

Of broad significance is that this phytoclime classification is underpinned by indicator taxa, suggesting that the phylogenetic and functional groups are confounded in African savannas. Indeed, floristic studies of African savannas suggest that tree community compositions are the outcome of phylogenetic filtering and not dispersal limitation^48^. This leads us to speculate that African savannas are dominated by a small set of taxonomic groups that were pre-adapted to savannas and radiated within the savanna environment to establish a priority effect that has slowed radiation and adaptation of other lineages in the savanna climate space. Analogous results were found by Ackerly in a more local study in the Californian chaparral^9^. This contrasts with evidence from other savanna systems such as the South American Cerrado, which showed rapid, in situ, convergent evolution of fire-related traits^60^. This study uses a simplified phylogenetic proxy for functionally relevant plant types and although it illustrates the potential of this approach, we acknowledge that further work is needed to expand this framework by potentially including other taxonomic groups that have been recognized to be indicators of different savannas^47,48^. Future studies should also explore phylogenetic relatedness explicitly by considering phylogenetic distance when fitting the species distribution models and building ecosystem-type classifications.

A more general challenge is to develop functional classifications that explicitly accommodate the tension between phylogenetic conservatism and evolutionary convergence. Currently, plant functional type and biome concepts assume that evolutionary convergence overwhelms phylogenetic niche conservatism and therefore that phylogenetic and biogeographical context can be ignored when forecasting Earth system dynamics. However, the idea of biome level convergence as an organizing principle for predicting global ecosystem properties has been questioned before, both in general^61^ and specifically for savannas^55^. As ecosystems reassemble under climate change^62^, it is likely that their near-term functional properties will be determined more by the traits of the taxonomic groups pre-adapted to these new climatic regimes than by the in situ convergent evolution of new adaptations. Our findings reinforce the view that biome-level convergence is not a universal organizing principle, and highlight the need to integrate phylogenetic context when predicting ecosystem properties under global change.

## Methods

### Approach

We identified phytoclimes - regions where climate favours the growth of similar combinations of plant types^14^, using plant types that are taxonomically defined. The foundation of this approach is laid by the assumption that we can use species distribution data to reveal the environmental preferences of different plant types^42^. We used the existing literature^15–19^ to identify meaningful plant types. As motivated in the main text we used seven savanna taxa to define the types. We use the term taxa here to refer to paraphyletic assemblages of species that belong to a specific taxon (family, subfamily or genus) and occur in African savannas. The taxa were *Vachellia* (Fabaceae); the genus *Commiphora* (Burseraceae); the family Combretaceae; the subfamily Detarioideae (Fabaceae); the subfamilies Panicoideae, Chloridoideae and Aristidoideae of the Poaceae. The workflow involved first fitting a species distribution model to all species with sufficient data of each group and then averaging the suitability scores of all member species at each location. The seven resulting taxonomic group suitability surfaces were then clustered into classes, and these classes represent the phytoclimes. In the next sections we describe the details. All analyses were performed using the statistical software environment R^63^, while maps were created using the R package terra^64^.

### Plant growth model for species distribution modelling

In principle, any species distribution model can be used to estimate the climatic suitability of geographic locations for the plant groups of interest. We have chosen a process-based plant growth model^65^ as implemented in the R package TTR.PGM^44^ that infers how climatic factors define the physiological limits of plant distributions. The underlying physiological model^43^ simulates Carbon (C) and Nitrogen (N) allocation between shoot and root and how these substrate pools determine biomass growth. Physiological rates in the model (growth, C and N assimilation) are constrained by the environment; and it is assumed that these constraints are species specific^65^. We used a 15 parameter version of the OAK variant of the model^44^.

### Environmental data for the plant growth model

The forcing data for fitting the physiological model was derived from a range of 1 km^2^ data products. The CHELSA 2.1 ambient dataset (1981–2010^66^) was used for monthly averages of daily mean, minimum and maximum air temperature, solar radiation, potential evapotranspiration and precipitation. A soil water balance model included in the TTR.PGM package was used to calculate the monthly soil water content using the CHELSA 2.1 precipitation, radiation and potential evapotranspiration data along with soil wilting point and field capacity data^67^. A Farquhar type photosynthesis model^68^ included in the TTR.PGM package was used to calculate C3 and C4 photosynthesis rates as forced by atmospheric CO_2_, air temperature, and solar radiation. The atmospheric CO_2_ concentration was assumed to be a constant 338 ppm.

### Species data for the plant growth model

We used presence- and absence geographical data of 1255 savanna species to estimate species-level physiological parameters in the model. The species data covered the African continent with small gaps (Supplementary Fig. S4). Species were chosen as follows: A species list provided for the African continent by BIEN^69^ was filtered for those species that belonged to the selected African savanna taxonomic groups (i.e., *Vachellia*; Detarioideae; Combretaceae; *Commiphora*; Panicoideae; Chloridoideae; Aristidoideae). Further, species had to have at least two occurrences in the savanna region of the White map ^19^ (Supplementary Fig. S1). The savanna region in Africa as outlined by White^19^ includes, in White’s terminology, woodland, wooded grassland and bushland (map units 22, 25–32, 35–37 & 40–48). True forest species in Detarioideae and Combretaceae were further filtered out using the AFROTROPTREE dataset^70^ if they were present in a plot delimited in that dataset as “forest” as our goal was to specifically determine savanna taxonomic group suitability. We acknowledge that the taxonomic groups used in our study do not exclusively occur in African savannas. Some heavily cultivated species were preemptively removed from the species list (*Zea mays*, *Sorghum* sp., *Tamarindus indica*). Climbers in Combretaceae were excluded using the BIEN trait database^69,71^ and the RAINBIO database^72^. Taxonomic resolution was performed using the taxonomic name resolution service (TNRS) provided by BIEN^73^. We originally considered Acacia s.l. (including *Senegalia* next to *Vachellia*) but in the final analyses use only *Vachellia* as results were qualitatively similar and Acacia s.l. is not monophyletic.

The occurrence data for each model fit was a geographically homogenous sample of ca. 100 presence, ca. 100 true absence and ca. 100 pseudo-absence points to inversely estimate physiological response curves for each species. The three different data types were handled as follows: Presence locations for each species were derived from geo-referenced vouchered specimens from GBIF^74,75^, and the RAINBIO database for Africa^72^. Records were filtered using the R Package CoordinateCleaner^76^, removing records close to country centroids, capitals, biological institutions or botanical gardens. Presence data was spatially thinned to a target number of 100 using the R package SpThin^77^. Next to spatial thinning, we also performed a post-hoc analysis to ensure our model residuals are not substantially autocorrelated (Supplementary Fig. S5). For this, we calculated Moran’s I with the R package terra^64^ for the Pearson residuals of all 1255 model fits, where Pearson residuals were calculated using Eq. (1):

1 $${r}_{i}=\frac{y-\widehat{y}}{\sqrt{\widehat{y}\times (1-\widehat{y})}}$$

where *y* was the observed presence-absence data for a fit *i* and $\widehat{y}$ was the predicted probability of occurrence.

Plot data was used to further add presence locations to the presence-only data. Additionally, plot data was used as true absence data for a species in locations where it was missing. Plot data used in this study was: the AFROTROPTREE database^70^, which is a collection of species lists from 753 afrotropical forest and savanna sites; National Vegetation Database of South Africa (NVD) and associated ACKDAT database, which is a digitization of South African vegetation mapping efforts spearheaded by John Acocks^23,78,79^, the West African Vegetation database^80^ by Senckenberg BiK-F that provided around 1200 locations in west Africa, originally stemming from the SUN and BIOTA projects; the FLOTROP database^81^, which contributed around 11,000 locations from the Sudano-Sahel area of Africa. The West African Vegetation and FLOTROP databases were accessed via GBIF^74^, these occurrences were filtered for full inventory surveys and species lists created for each unique geolocation. The providers of both databases noted that some surveys only included woody species, herbaceous species or both. This information was not explicitly given in the database survey details. Therefore, plots from both databases were classified as “tree only”, “herb only” or “both” using life form data from the BIEN^69,71^ and RAINBIO databases^72^. This classification was then used to reliably derive absence data for each life form, e.g., omitting “herb only” locations for tree species absence sampling. This pool of absence locations was then used to sample true absence locations for each species. Additionally, pseudo-absence points were created. The creation of pseudo-absence was stratified such that Koeppen-Geiger climate zones not yet represented in true presence and absence points were sampled. This ensured that the entire global environmental range was represented in the sample. The sampling of absence points was structured so that the final data set for a species consisted of 1/3 of each presence, true-absence, and pseudo-absence data points.

### Parametrization of the plant growth model

Models were only attempted if the previous step produced data sets with 5 or more presence records (i.e. 15 or more presence-absence points). 97.4% of species fits had more than 5 and 89.0% of fits more than 10 presence data points (and thus more than 15 or 30 total data points, respectively). Species data sources are documented in Supplementary Table S7. Photosynthetic parameters of the Farquhar-type submodel were assumed to be constant across species save for photosynthetic type (C_3_ vs. C_4_). All tree species were assumed to be C_3_, while literature was consulted to determine photosynthetic type for grasses^82^.

We generated initial parameter estimates using the differential evolution genetic algorithm (as implemented in the R package DEoptim^83^). These parameter estimates where use to initiate an MCMC-based algorithm (random walk Metropolis) using the R package LaplacesDemon^84^. The posterior parameter estimates were then used for a projection of the model-estimated probability of occurrence for a given species in geographic space. We generated species distribution models for 1255 species. Modelled geographic distributions were evaluated with measures of predictive accuracy, the area under curve (AUC) and the true skill statistic (TSS; Supplementary Table S1). Typical threshold values for identifying poor fits from studies using the TTR.PGM for phytoclime identification were adapted from previous work^14,42^. These studies used AUC thresholds of >0.75 and TSS thresholds of >0.7. Using the AUC threshold, 99.6% of attempted fits were retained, whereas only 78.9% are retained under the TSS threshold. We used the AUC threshold because our subsequent steps in the analysis were not sensitive to this decision (Supplementary Fig. S6).

### Suitability and phytoclime determination

The remaining 1248 range maps were overlaid and averaged to create one geographic suitability surface per phylogenetic group. The suitability surfaces were then normalized by dividing the values of all locations by the maximum suitability value, thus creating a relative suitability surface for each group ranging between 0 and 1. Phytoclimes were identified by applying an unsupervised model-based clustering algorithm implemented in the R package mclust^85^ to the seven suitability surfaces, i.e., clustering sites according to the site × taxonomic group suitability matrix for the seven taxonomic groups. The geographic region used in this classification was restricted to the savanna region delimited by White^19^. A moderate number of clusters, seven, was selected to balance goodness-of-fit of the clustering model and ecological interpretability (see Supplementary Fig. S7 for goodness-of-fit for 1–30 clusters). For transparency, we provide phytoclime maps for cluster numbers 2–10, as there is no substantial improvement of model fit for more than 10 clusters (Supplementary Fig. S8).

### Formal map comparison

The phytoclime map was quantitatively compared to the White map (ref. ^19^, Supplementary Fig. S1). We achieved this by following a protocol for formal comparison of two maps with differing categories as described in ref. ^45^. The “major vegetation types and mosaics”-categories from the savanna region of White’s map (cf. Supplementary Table S8) were used. What constituted the savanna region on White’s map for this analysis is described in the section “Species data”. The number of categories on the maps were harmonized by computing the overlap of mapping categories on a pixel level and merging categories that shared the same majority of overlap (see Supplementary Table S8 and ref. ^45^ for details). A contingency table was then computed (see ref. ^45^). Cohen’s kappa (*κ*), a measure of agreement, was calculated for each pair of map categories using Eq. (2):

2 $$\kappa =\frac{{p}_{ij}-{p}_{i}{p}_{j}}{\frac{{p}_{i}+{p}_{j}}{2}-{p}_{i}{p}_{j}}$$

where *p*_*ij*_ is the observed fraction of pixels in which category *i* (from map 1) and category *j* (from map 2) coincide. The terms *p*_*i*_ and *p*_*j*_ are the proportions of the respective maps occupied by categories *i* and *j*. In words, the numerator expresses how much the observed coincidence (*p*_*ij*_) exceeds the coincidence expected by chance (*p*_*i*_*p*_*j*_). The denominator scales this difference by the maximum possible coincidence given the category prevalences $\left(\frac{{p}_{i}+{p}_{j}}{2}\right)$, adjusted for the chance expectation. Then agreement was mapped by displaying Cohen’s *κ* in geographic space for each pairing of categories at a given pixel. Cohen’s *κ* gives a value ranging between −1 and 1 where, according to existing conventions ^86^, *κ* ≤0.40 indicates poor agreement, 0.40 < *κ* < 0.75 indicates good agreement, and *κ* ≥0.75 indicates very good agreement. The same workflow was also applied to compare the agreement of a growth-form-defined mapping of African biomes^42^ with the White map (see Supplementary Table S9).

### Post-hoc analyses

We analysed the phytoclime outputs using recursive partitioning as implemented in the R package rpart^87^. We produced a classification tree that predicted phytoclimes from mean annual temperature (^∘^C) and effective rainfall (mm) and a second tree that predicted phytoclimes from the savanna taxonomic group suitability surfaces generated from the species distribution models (Fig. 1). The recursive partitioning produces a classification tree for the given dataset and finds a solution in the predictor matrix (site × climate or site × taxonomic suitability matrix) for each split that best reduces the Gini impurity^87^. Effective rainfall was defined as mean annual rainfall - evapotranspiration. For this analysis, evapotranspiration and temperature data were taken from CHELSA 2.1^66^. The control parameter in rpart was manually adjusted for each fit to yield acceptable cross-validated error rates. The R package rpart.plot^88^ was used to plot the resulting regression trees.

### Functional attributes of the phytoclimes

To explore functional differences between the phytoclimes we examined their net primary production, fire activity and ability to support herbivores. Net primary productivity (NPP) was extracted from the annual MODIS NPP data product (MOD17A3HGF^89^) using data from 2001 to 2023. For NPP, only protected areas were considered to exclude effects of land-use. Information on protected areas in Africa was taken from the World protected area database^90^. NPP values above 3.0 were excluded, as they were considered ecologically unrealistic for savannas and to be artifacts of land-use or the remote sensing analysis. The density of the major livestock species in Africa (cattle, *Bos taurus*) was extracted from the UN-FAO gridded livestock census data product of 2020^59^. This was taken as a proxy of how much livestock herbivory is supported by the different savanna types, which is expected to be a function of forage amount and forage quality. Fire activity was compared using mean annual burnt area and the coefficient of variation of annual burnt area (as an indicator of return interval) from the Global Fire Emissions dataset 4.1^91^. This dataset offers burnt area [% of pixel] on an annual temporal resolution with coverage of 19 years between 1997 and 2015. These data products were sampled at the resolution of the phytoclime map (0.25 × 0.25 degrees) for each phytoclime.

The ability to predict variation in these ecosystem properties using the taxa-based phytoclime classification was formally analysed and compared to an existing growth-form-based phytoclime classification^42^, as well as the floristic map published by White^19^ (Supplementary Fig. S3, Supplementary Tables S2, S3, S5, S6). We fitted Bayesian linear models in JAGS^92^, with the ecosystem metrics as response variables and the phytoclime classes as predictor variables. Priors were uninformed uniform. The predictive performance of the models was evaluated using the Widely Applicable Information Criterion (WAIC) and leave-one-out cross-validation (LOO) as implemented in the R package loo^93^. Absolute goodness-of-fit was further assessed using the Gelman’s R2 statistic^94^. Both WAIC and LOO provide measures of out-of-sample predictive accuracy that account for model complexity. Lower WAIC values and higher (less negative) LOO values indicate better predictive performance.

## Supplementary information

Supplementary Information

## Data Availability

The study was conducted using publicly available datasets as outlined in the methods sections. A comprehensive list of the used species is provided in Supplementary Table S1. We will provide the presence-absence dataset derived from Supplementary Table S7 used for species distribution modelling for transparency as data sources might have slightly changed or public availability might have become restricted in a public repository (10.5281/zenodo.20269856).

## Code availability

Analyses were conducted in R^63^, an openly accessible statistical environment using publicly available extensions, R packages, as outlined in the methods section. Code for fitting the species distribution model, as well as analysis and figure generation will be publicly reposited (10.5281/zenodo.20269856).
