# Supplementary material for: Climatic preferences of major plant clades define the functional attributes of African savanna types
Source: NPJ Biodivers. 2026 May 23;5:20. doi: 10.1038/s44185-026-00138-5 (PMC13269999; doi:10.1038/s44185-026-00138-5)
Supplement: Supplementary file 1 — Supplementary Information [file 44185_2026_138_MOESM1_ESM.pdf]

Supplementary material for: Climatic preferences of major plant clades define the functional attributes of African savanna types

Vitus Pickelmann, Steven I. Higgins

April 29, 2026

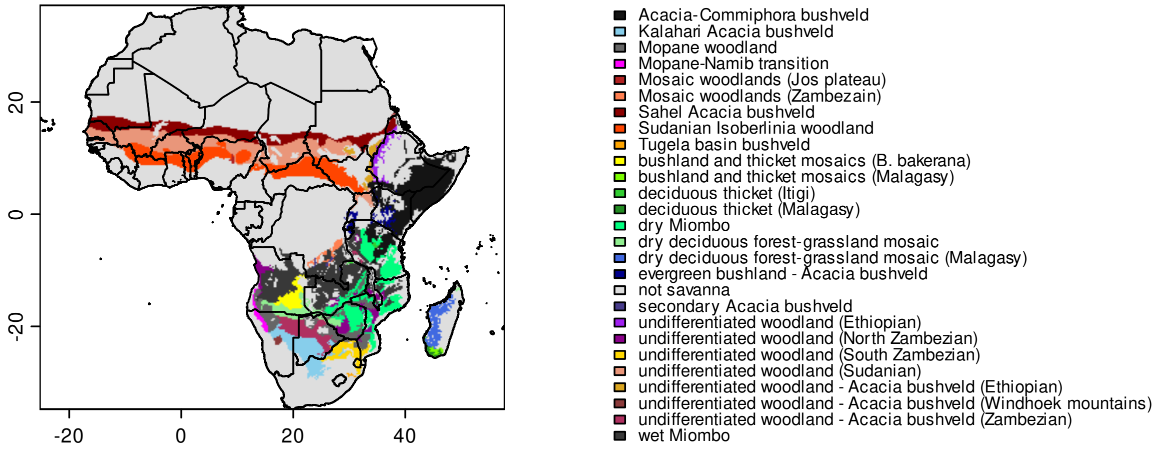

Figure S1: Map used for formal comparison with the phytoclimate map generated in this study. The map considers only the savanna types of the White Vegetation map [1]. Gray areas indicate non-savanna areas

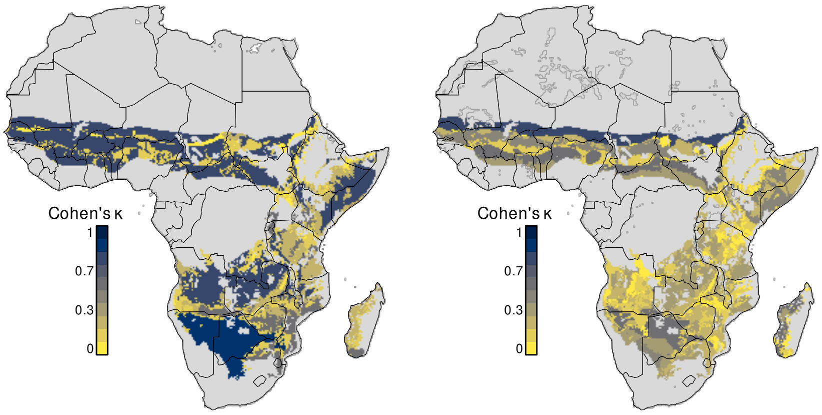

Figure S2: Comparison of ability of a phylogenetically based classification (manuscript Fig. 1, left) and a growth form based classification [2] (right) to predict the map classes proposed by White [1] (Fig. S1). The agreement was estimated using Cohen's κ. Existing conventions [3] interpret $\kappa \leq 0.40$ as poor agreement, $0.40 < \kappa < 0.75$ as good agreement and $\kappa \geq 0.75$ as very good agreement. Gray areas on both maps indicate non-savanna areas.

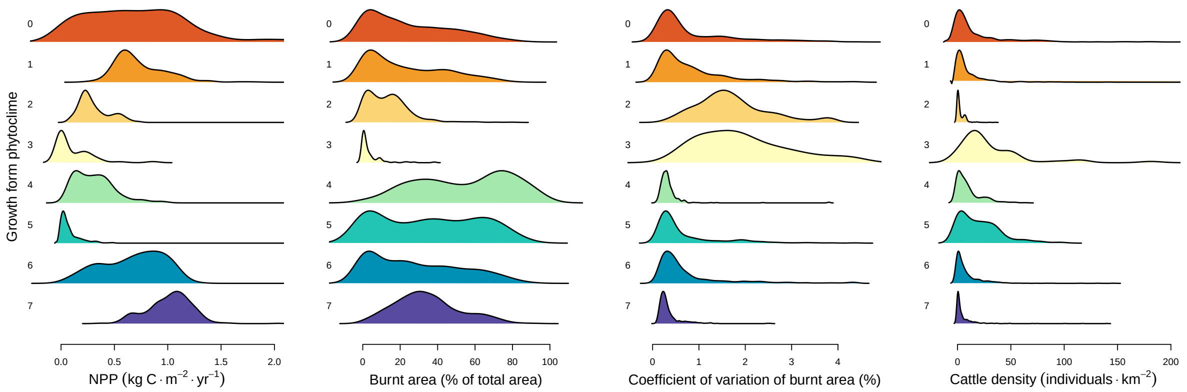

Figure S3: Kernel density estimates of functional attributes of phytoclimes defined using plant growth forms from a previous study [2].

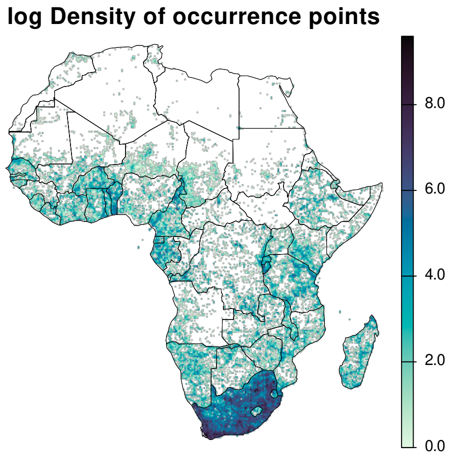

Figure S4: Spatial coverage of the species data. log Density of occurrence points per projected phytoclime pixel is shown across the African continent. Transparent pixels had no occurrence points. This shows all species data, the data used to fit individual models was spatially thinned.

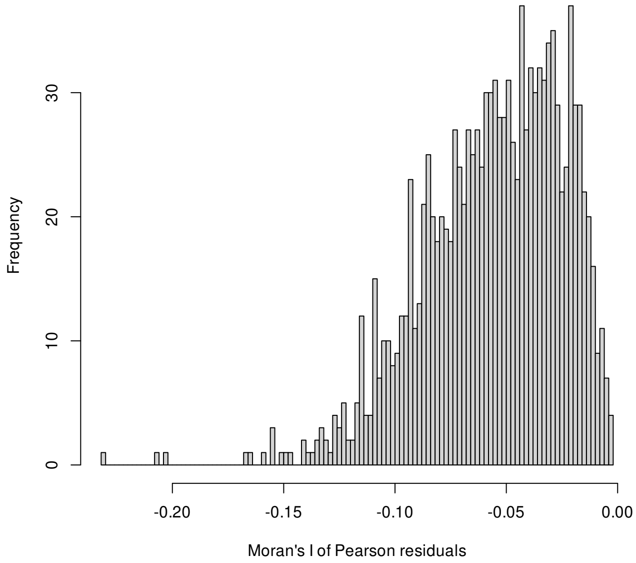

Figure S5: Histogram of Moran's I for individual species model Pearson residuals. This shows potential residual spatial autocorrelation that could not be accounted for by spatially thinning occurrence points. 0 indicates spatial independence. Moran's I was calculated using the residuals of each of the 1255 species fits. The exact calculation is outlined in the Methods section.

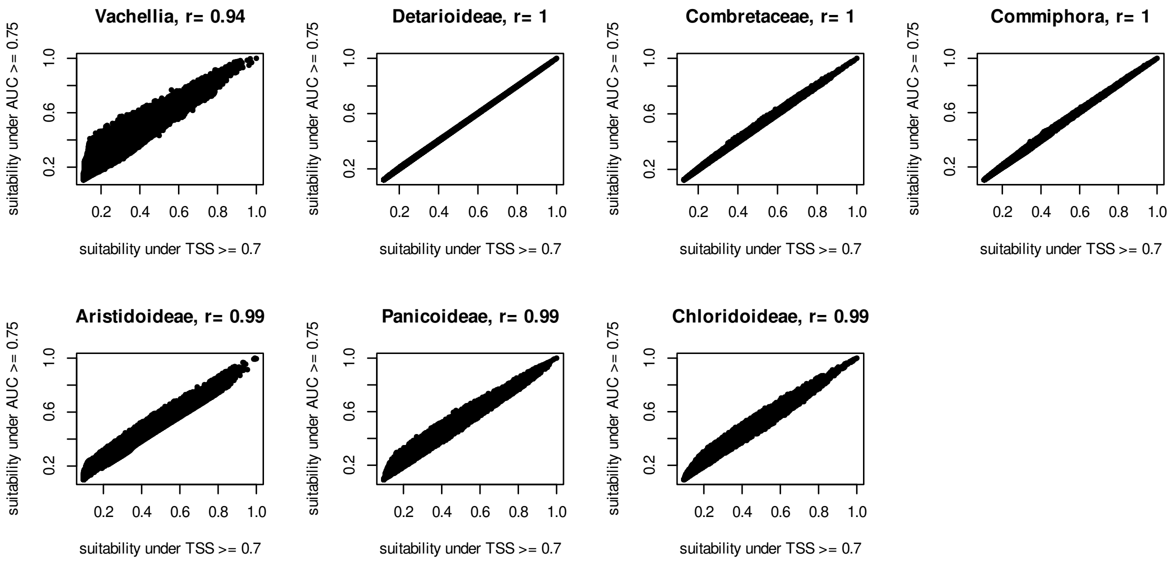

Figure S6: Correlation of suitability surface values when using different thresholds to determine goodness-of-fit.

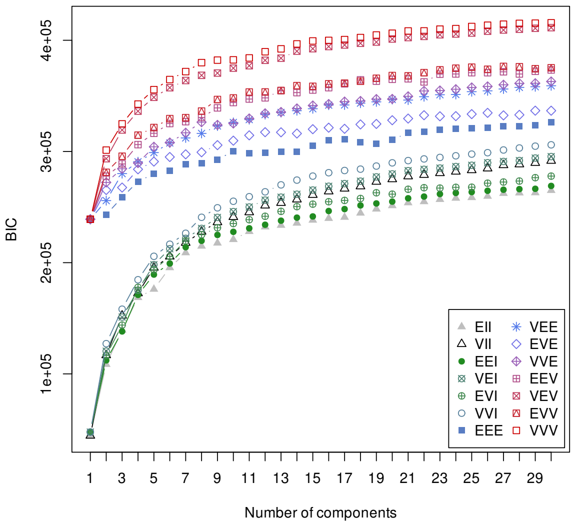

Figure S7: Model comparison (goodness-of-fit) from the model-based clustering using [4] for 1-30 clusters using the BIC, where higher values indicate a better goodness-of-fit. Different symbols/colours indicate different model setups as outlined in [4].

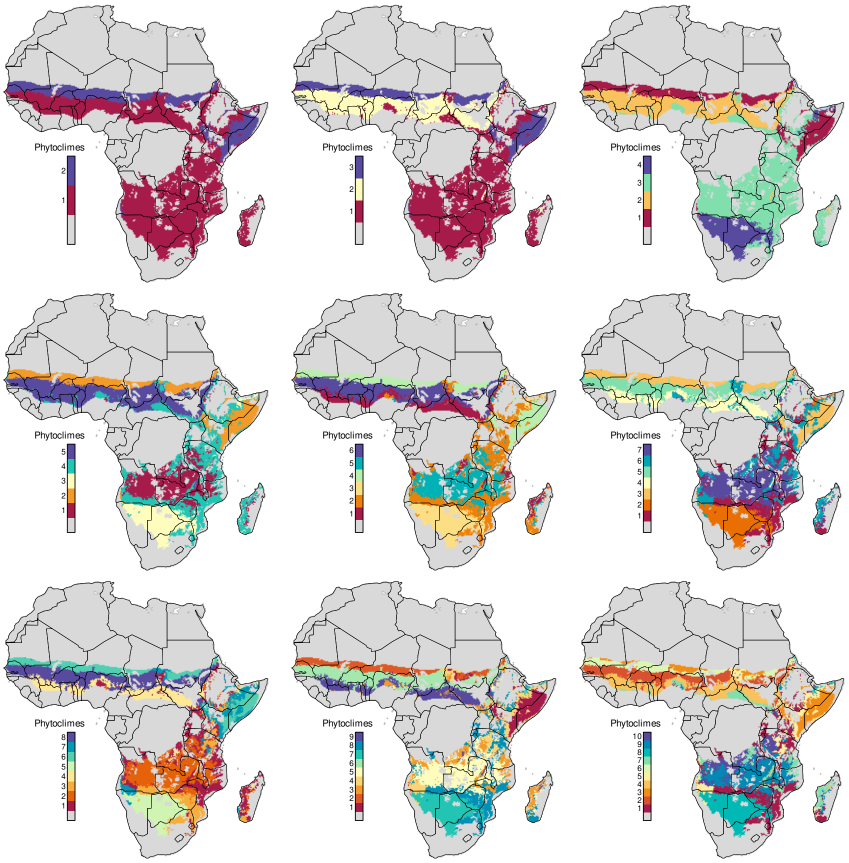

Figure S8: Maps of the phytoclimes for different cluster numbers other than seven.

Table S1: Full list of attempted species distribution models. Evaluation metrics are given for each species: TSS = True skill statistic, AUC = Area under ROC. Acceptable models had to have AUC >= 0.75; those omitted marked with "O-" in the enumeration column of the table. Taxonomic group association is also given. Note that *Senegalia* was not considered in the final analysis, but is included for transparency and posterity.

	species name	TSS	AUC	group
1	Acrachne perrieri	0.91	0.98	Chloridoideae
2	Acrachne racemosa	0.70	0.87	Chloridoideae
3	Acroceras amplectens	0.75	0.87	Panicoideae
4	Acroceras attenuatum	0.96	0.97	Panicoideae
5	Acroceras boivinii	0.80	0.89	Panicoideae
6	Acroceras calcicola	0.77	0.91	Panicoideae
7	Acroceras gabunense	0.86	0.96	Panicoideae
8	Acroceras hubbardii	0.87	0.96	Panicoideae
9	Acroceras macrum	0.74	0.92	Panicoideae
10	Acroceras zizanioides	0.75	0.93	Panicoideae
11	Adenochloa adenophora	0.86	0.95	Panicoideae
12	Afzelia peturei	0.94	0.96	Detarioideae
13	Afzelia quanzensis	0.75	0.92	Detarioideae
14	Alloeochaete namuliensis	1.00	1.00	Panicoideae
15	Alloeochaete oreogena	0.97	0.98	Panicoideae
16	Anadelphia afzeliana	0.79	0.90	Panicoideae
17	Anadelphia leptocoma	0.84	0.97	Panicoideae
18	Anadelphia liebigiana	0.89	0.90	Panicoideae
19	Anadelphia scyphofera	0.97	0.97	Panicoideae
20	Andropogon africanus	0.78	0.92	Panicoideae
21	Andropogon amethystinus	0.78	0.93	Panicoideae
22	Andropogon appendiculatus	0.83	0.95	Panicoideae
23	Andropogon auriculatus	0.79	0.93	Panicoideae
24	Andropogon brazzae	0.77	0.90	Panicoideae
25	Andropogon canaliculatus	0.73	0.90	Panicoideae
26	Andropogon chevalieri	0.90	0.96	Panicoideae
27	Andropogon chinensis	0.54	0.81	Panicoideae
28	Andropogon chrysostachyus	0.86	0.94	Panicoideae
29	Andropogon curvifolius	0.84	0.93	Panicoideae
30	Andropogon distachyos	0.76	0.94	Panicoideae
31	Andropogon eucomus	0.71	0.89	Panicoideae
32	Andropogon festuciformis	0.77	0.94	Panicoideae
33	Andropogon gabonensis	0.81	0.95	Panicoideae
34	Andropogon gayanus	0.59	0.84	Panicoideae
35	Andropogon greenwayi	0.68	0.88	Panicoideae
36	Andropogon ibityensis	0.97	0.98	Panicoideae
37	Andropogon ivohibensis	0.72	0.88	Panicoideae
38	Andropogon ivorensis	0.82	0.93	Panicoideae
39	Andropogon kelleri	0.90	0.98	Panicoideae
40	Andropogon lacunosus	0.90	0.98	Panicoideae
41	Andropogon ligulatus	0.99	1.00	Panicoideae
42	Andropogon lima	0.90	0.98	Panicoideae
43	Andropogon macrophyllus	0.89	0.96	Panicoideae
44	Andropogon mannii	0.76	0.90	Panicoideae
45	Andropogon perligulatus	0.76	0.91	Panicoideae
46	Andropogon pinguipes	0.86	0.95	Panicoideae
47	Andropogon pseudapricus	0.75	0.90	Panicoideae
48	Andropogon pteropholis	0.94	0.99	Panicoideae
49	Andropogon schirensis	0.63	0.88	Panicoideae
50	Andropogon tectorum	0.79	0.92	Panicoideae
51	Andropogon tenuiberbis	0.81	0.90	Panicoideae
52	Andropogon virgatus	0.80	0.94	Panicoideae
53	Andropterum stolzii	0.89	0.96	Panicoideae
54	Aphanocalyx richardsiae	0.80	0.95	Detarioideae
55	Aphanocalyx trapnellii	0.94	0.99	Detarioideae
56	Apochiton burttii	0.96	0.99	Chloridoideae
57	Aristida adoensis	0.73	0.90	Aristidoideae
O-58	Aristida adscensionis	0.36	0.74	Aristidoideae
59	Aristida aequiglumis	0.94	0.98	Aristidoideae
60	Aristida ambongensis	0.75	0.84	Aristidoideae
61	Aristida barbicollis	0.81	0.94	Aristidoideae
62	Aristida bipartita	0.91	0.98	Aristidoideae
63	Aristida congesta	0.76	0.93	Aristidoideae
64	Aristida cumingiana	0.70	0.87	Aristidoideae
65	Aristida diffusa	0.80	0.94	Aristidoideae
66	Aristida diminuta	0.76	0.86	Aristidoideae
67	Aristida engleri	0.91	0.98	Aristidoideae

	species name	TSS	AUC	group
68	Aristida funiculata	0.62	0.86	Aristidoideae
69	Aristida holathera	0.77	0.94	Aristidoideae
70	Aristida hordeacea	0.59	0.83	Aristidoideae
71	Aristida hubbardiana	0.95	0.98	Aristidoideae
72	Aristida junciformis	0.69	0.89	Aristidoideae
73	Aristida kelleri	0.77	0.93	Aristidoideae
74	Aristida kenyensis	0.76	0.93	Aristidoideae
75	Aristida kerstingii	0.83	0.94	Aristidoideae
76	Aristida leucophaea	0.77	0.89	Aristidoideae
77	Aristida lisowskii	1.00	1.00	Aristidoideae
78	Aristida meridionalis	0.82	0.96	Aristidoideae
79	Aristida mollissima	0.87	0.98	Aristidoideae
80	Aristida mutabilis	0.60	0.82	Aristidoideae
81	Aristida nemorivaga	0.86	0.97	Aristidoideae
82	Aristida paoliana	0.96	0.98	Aristidoideae
83	Aristida parvula	0.83	0.96	Aristidoideae
84	Aristida pilgeri	0.91	0.96	Aristidoideae
85	Aristida recta	0.72	0.90	Aristidoideae
86	Aristida rhiniochloa	0.81	0.92	Aristidoideae
87	Aristida rufescens	0.81	0.90	Aristidoideae
88	Aristida scabrivalvis	0.87	0.96	Aristidoideae
89	Aristida sciurus	0.93	0.99	Aristidoideae
90	Aristida sieberiana	0.67	0.86	Aristidoideae
91	Aristida similis	0.82	0.94	Aristidoideae
92	Aristida spectabilis	0.86	0.95	Aristidoideae
93	Aristida stenophylla	0.81	0.94	Aristidoideae
94	Aristida stenostachya	0.75	0.92	Aristidoideae
95	Aristida stipitata	0.84	0.94	Aristidoideae
96	Aristida stipoides	0.75	0.88	Aristidoideae
97	Aristida tenuissima	0.93	0.98	Aristidoideae
98	Aristida transvaalensis	0.92	0.97	Aristidoideae
99	Aristida triticoides	0.77	0.93	Aristidoideae
100	Aristida vestita	0.80	0.95	Aristidoideae
101	Arthraxon hispidus	0.73	0.90	Panicoideae
102	Arthraxon lanceolatus	0.68	0.91	Panicoideae
103	Arthraxon lancifolius	0.67	0.87	Panicoideae
104	Arthraxon prionodes	0.72	0.90	Panicoideae
105	Arundinella nepalensis	0.63	0.81	Panicoideae
106	Baikiaea plurijuga	0.94	0.98	Detarioideae
107	Berlinia giorgii	0.84	0.95	Detarioideae
108	Berlinia sapinii	0.85	0.91	Detarioideae
109	Bewsia biflora	0.76	0.92	Chloridoideae
110	Bothriochloa bladhii	0.55	0.81	Panicoideae
111	Bothriochloa insculpta	0.62	0.88	Panicoideae
112	Bothriochloa pertusa	0.63	0.86	Panicoideae
113	Bothriochloa radicans	0.79	0.94	Panicoideae
114	Brachiaria ambigens	0.86	0.94	Panicoideae
115	Brachiaria bemarivensis	0.76	0.90	Panicoideae
116	Brachiaria dimorpha	0.91	0.99	Panicoideae
117	Brachiaria fragrans	0.77	0.94	Panicoideae
118	Brachiaria glomerata	0.78	0.93	Panicoideae
119	Brachiaria grossa	0.84	0.95	Panicoideae
120	Brachiaria humbertiana	0.83	0.90	Panicoideae
121	Brachiaria marlothii	0.89	0.98	Panicoideae
122	Brachiaria nana	0.82	0.94	Panicoideae
123	Brachiaria perrieri	0.90	0.94	Panicoideae
124	Brachiaria pseudodichotoma	0.82	0.90	Panicoideae
125	Brachiaria tsiafajavonensis	0.89	0.95	Panicoideae
126	Brachiaria umbellata	0.83	0.95	Panicoideae
127	Brachiaria umbratilis	0.91	0.99	Panicoideae
128	Brachychloa fragilis	0.95	0.99	Chloridoideae
129	Brachystegia allenii	0.92	0.98	Detarioideae
130	Brachystegia angustistipulata	0.93	0.96	Detarioideae
131	Brachystegia bakeriana	0.92	0.97	Detarioideae
132	Brachystegia boehmii	0.81	0.93	Detarioideae
133	Brachystegia bussei	0.79	0.94	Detarioideae
134	Brachystegia floribunda	0.86	0.94	Detarioideae
135	Brachystegia glaberrima	0.93	0.98	Detarioideae
136	Brachystegia gossweileri	0.94	0.98	Detarioideae
137	Brachystegia longifolia	0.83	0.94	Detarioideae
138	Brachystegia manga	0.80	0.93	Detarioideae
139	Brachystegia puberula	0.94	0.98	Detarioideae

	species name	TSS	AUC	group
140	Brachystegia stipulata	0.88	0.95	Detarioideae
141	Brachystegia tamarindoides	0.77	0.93	Detarioideae
142	Brachystegia taxifolia	0.95	0.98	Detarioideae
143	Brachystegia utilis	0.77	0.92	Detarioideae
144	Brandzeia filicifolia	0.91	0.98	Detarioideae
145	Bromuniola gossweileri	0.89	0.97	Panicoideae
146	Capillipedium parviflorum	0.67	0.88	Panicoideae
147	Cenchrus americanus	0.53	0.80	Panicoideae
148	Cenchrus biflorus	0.52	0.79	Panicoideae
149	Cenchrus caudatus	0.62	0.87	Panicoideae
150	Cenchrus ciliaris	0.55	0.78	Panicoideae
151	Cenchrus clandestinus	0.68	0.88	Panicoideae
152	Cenchrus echinatus	0.59	0.86	Panicoideae
153	Cenchrus geniculatus	0.79	0.94	Panicoideae
154	Cenchrus hordeoides	0.79	0.93	Panicoideae
155	Cenchrus longisetus	0.72	0.91	Panicoideae
156	Cenchrus massaicus	0.74	0.90	Panicoideae
157	Cenchrus mitis	0.68	0.88	Panicoideae
158	Cenchrus pedicellatus	0.60	0.84	Panicoideae
159	Cenchrus pennisetiformis	0.74	0.88	Panicoideae
160	Cenchrus prieurii	0.79	0.94	Panicoideae
161	Cenchrus procerus	0.83	0.96	Panicoideae
162	Cenchrus purpureus	0.69	0.90	Panicoideae
163	Cenchrus ramosus	0.64	0.83	Panicoideae
164	Cenchrus setaceus	0.60	0.87	Panicoideae
165	Cenchrus setigerus	0.69	0.88	Panicoideae
166	Cenchrus sieberianus	0.77	0.88	Panicoideae
167	Cenchrus sphacelatus	0.79	0.92	Panicoideae
168	Cenchrus stramineus	0.90	0.97	Panicoideae
169	Cenchrus trachyphyllus	0.86	0.96	Panicoideae
170	Cenchrus trisetus	0.88	0.95	Panicoideae
171	Cenchrus unisetus	0.70	0.87	Panicoideae
172	Cenchrus violaceus	0.72	0.90	Panicoideae
173	Centropodia glauca	0.84	0.94	Chloridoideae
174	Centropodia mossamedensis	0.62	0.86	Chloridoideae
175	Chasmopodium caudatum	0.76	0.90	Panicoideae
176	Chloris amethystea	0.80	0.90	Chloridoideae
177	Chloris barbata	0.64	0.86	Chloridoideae
178	Chloris gayana	0.56	0.81	Chloridoideae
179	Chloris humbertiana	0.90	0.95	Chloridoideae
180	Chloris mossambicensis	0.84	0.97	Chloridoideae
181	Chloris pilosa	0.56	0.82	Chloridoideae
182	Chloris pycnothrix	0.65	0.89	Chloridoideae
183	Chloris robusta	0.83	0.95	Chloridoideae
184	Chloris virgata	0.48	0.77	Chloridoideae
185	Chrysochloa hindsii	0.76	0.91	Chloridoideae
186	Chrysochloa hubbardiana	0.83	0.89	Chloridoideae
187	Chrysochloa orientalis	0.82	0.96	Chloridoideae
188	Chrysopogon aciculatus	0.76	0.93	Panicoideae
189	Chrysopogon aucheri	0.51	0.80	Panicoideae
190	Chrysopogon fulvibarbis	0.81	0.96	Panicoideae
191	Chrysopogon fulvus	0.70	0.83	Panicoideae
192	Chrysopogon nigritanus	0.65	0.85	Panicoideae
193	Chrysopogon plumulosus	0.55	0.84	Panicoideae
194	Chrysopogon serrulatus	0.72	0.88	Panicoideae
195	Chrysopogon zizanioides	0.73	0.88	Panicoideae
196	Cladoraphis cyperoides	0.87	0.97	Chloridoideae
197	Cleistachne sorghoides	0.82	0.94	Panicoideae
198	Colophospermum mopane	0.74	0.92	Detarioideae
199	Combretum acutifolium	0.95	0.97	Combretaceae
200	Combretum acutum	0.93	0.97	Combretaceae
201	Combretum albiflorum	0.82	0.93	Combretaceae
202	Combretum albopunctatum	0.90	0.97	Combretaceae
203	Combretum andradae	0.99	0.99	Combretaceae
204	Combretum apiculatum	0.66	0.89	Combretaceae
205	Combretum aureonitens	0.86	0.98	Combretaceae
206	Combretum bracteosum	0.85	0.95	Combretaceae
207	Combretum butyrosum	0.84	0.94	Combretaceae
208	Combretum camporum	0.76	0.95	Combretaceae
209	Combretum capituliflorum	0.67	0.87	Combretaceae
210	Combretum celastroides	0.80	0.93	Combretaceae
211	Combretum chionanthoides	0.94	0.99	Combretaceae

	species name	TSS	AUC	group
212	Combretum cinereopetalum	0.90	0.97	Combretaceae
213	Combretum collinum	0.67	0.87	Combretaceae
214	Combretum constrictum	0.76	0.93	Combretaceae
215	Combretum contractum	0.97	0.99	Combretaceae
216	Combretum coursianum	0.97	1.00	Combretaceae
217	Combretum decaryi	0.83	0.88	Combretaceae
218	Combretum elaeagnoides	0.86	0.96	Combretaceae
219	Combretum engleri	0.86	0.98	Combretaceae
220	Combretum erythrophyllum	0.82	0.95	Combretaceae
221	Combretum exalatum	0.75	0.91	Combretaceae
222	Combretum exannulatum	0.91	0.93	Combretaceae
223	Combretum falcatum	0.77	0.94	Combretaceae
224	Combretum fuscum	0.74	0.92	Combretaceae
225	Combretum gillettianum	0.99	1.00	Combretaceae
226	Combretum glutinosum	0.77	0.90	Combretaceae
227	Combretum goetzei	0.89	0.95	Combretaceae
228	Combretum gossweileri	0.84	0.93	Combretaceae
229	Combretum grandidieri	0.86	0.97	Combretaceae
230	Combretum hartmannianum	0.52	0.76	Combretaceae
231	Combretum haullevilleanum	0.88	0.95	Combretaceae
232	Combretum hereroense	0.73	0.93	Combretaceae
233	Combretum holstii	0.92	0.96	Combretaceae
234	Combretum imberbe	0.69	0.89	Combretaceae
235	Combretum indicum	0.66	0.88	Combretaceae
236	Combretum kirkii	0.93	0.97	Combretaceae
237	Combretum kraussii	0.84	0.96	Combretaceae
238	Combretum lecardii	0.86	0.93	Combretaceae
239	Combretum longicollum	0.80	0.90	Combretaceae
240	Combretum longispicatum	0.84	0.96	Combretaceae
241	Combretum macrocalyx	0.81	0.96	Combretaceae
242	Combretum meridionalis	0.82	0.93	Combretaceae
243	Combretum micranthum	0.73	0.87	Combretaceae
244	Combretum microphyllum	0.70	0.90	Combretaceae
245	Combretum mkuzense	0.88	0.96	Combretaceae
246	Combretum moggii	0.77	0.93	Combretaceae
247	Combretum mossambicense	0.71	0.90	Combretaceae
248	Combretum nigricans	0.80	0.91	Combretaceae
249	Combretum nioroense	0.86	0.94	Combretaceae
250	Combretum nusbaumeri	0.96	0.96	Combretaceae
251	Combretum obovatum	0.82	0.95	Combretaceae
252	Combretum obscurum	0.71	0.90	Combretaceae
253	Combretum oxystachyum	0.78	0.90	Combretaceae
254	Combretum padoides	0.72	0.91	Combretaceae
255	Combretum pecoense	0.89	0.97	Combretaceae
256	Combretum pentagonum	0.79	0.93	Combretaceae
257	Combretum petrophilum	0.87	0.96	Combretaceae
258	Combretum pisoniiflorum	0.71	0.89	Combretaceae
259	Combretum psidioides	0.76	0.92	Combretaceae
260	Combretum purpureiflorum	0.89	0.98	Combretaceae
261	Combretum schumannii	0.74	0.92	Combretaceae
262	Combretum sericeum	0.75	0.91	Combretaceae
263	Combretum subglabratum	0.98	0.99	Combretaceae
264	Combretum subumbellatum	0.81	0.93	Combretaceae
265	Combretum tomentosum	0.80	0.91	Combretaceae
266	Combretum umbricola	0.79	0.90	Combretaceae
267	Combretum vendae	0.83	0.97	Combretaceae
268	Combretum villosum	0.91	0.98	Combretaceae
269	Combretum wattii	0.92	0.95	Combretaceae
270	Combretum xanthothyrsum	0.90	0.99	Combretaceae
271	Combretum zeyheri	0.75	0.92	Combretaceae
272	Commiphora acuminata	0.66	0.89	Commiphora
273	Commiphora africana	0.46	0.79	Commiphora
274	Commiphora anacardiifolia	0.76	0.91	Commiphora
275	Commiphora angolensis	0.88	0.95	Commiphora
276	Commiphora ankaranensis	0.99	1.00	Commiphora
277	Commiphora aprevalii	0.80	0.95	Commiphora
278	Commiphora arafy	0.95	0.98	Commiphora
279	Commiphora baluensis	0.82	0.96	Commiphora
280	Commiphora brevicalyx	0.80	0.90	Commiphora
281	Commiphora caerulea	0.76	0.94	Commiphora
282	Commiphora campestris	0.67	0.92	Commiphora
283	Commiphora capuronii	0.85	0.94	Commiphora

	species name	TSS	AUC	group
284	Commiphora chiovendana	0.84	0.90	Commiphora
285	Commiphora coleopsis	0.93	0.98	Commiphora
286	Commiphora confusa	0.91	0.96	Commiphora
287	Commiphora crenatoserrata	0.98	0.99	Commiphora
288	Commiphora dinteri	0.95	0.99	Commiphora
289	Commiphora edulis	0.75	0.93	Commiphora
290	Commiphora elliptica	0.98	0.99	Commiphora
291	Commiphora eminii	0.79	0.93	Commiphora
292	Commiphora engleri	0.96	0.97	Commiphora
293	Commiphora falcata	0.96	0.99	Commiphora
294	Commiphora franciscana	0.91	0.95	Commiphora
295	Commiphora fraxinifolia	0.89	0.95	Commiphora
296	Commiphora gileadensis	0.79	0.96	Commiphora
297	Commiphora glandulosa	0.78	0.94	Commiphora
298	Commiphora glaucescens	0.82	0.95	Commiphora
299	Commiphora gracilifrondosa	0.95	0.98	Commiphora
300	Commiphora grandifolia	0.89	0.98	Commiphora
301	Commiphora guillauminii	0.73	0.90	Commiphora
302	Commiphora gurreh	0.93	0.99	Commiphora
303	Commiphora harveyi	0.92	0.97	Commiphora
304	Commiphora hildebrandtii	0.81	0.97	Commiphora
305	Commiphora hornbyi	0.71	0.92	Commiphora
306	Commiphora humbertii	0.86	0.93	Commiphora
307	Commiphora kataf	0.76	0.94	Commiphora
308	Commiphora kerstingii	0.87	0.92	Commiphora
309	Commiphora kraeuseliana	0.84	0.92	Commiphora
310	Commiphora kua	0.68	0.93	Commiphora
311	Commiphora lamii	0.92	0.97	Commiphora
312	Commiphora lasiodisca	0.91	0.99	Commiphora
313	Commiphora laxecymigera	0.89	0.97	Commiphora
314	Commiphora leandriana	0.94	0.96	Commiphora
315	Commiphora madagascariensis	0.85	0.96	Commiphora
316	Commiphora mafaidoha	0.84	0.90	Commiphora
317	Commiphora mahafaliensis	0.98	0.99	Commiphora
318	Commiphora marchandii	0.85	0.95	Commiphora
319	Commiphora marlothii	0.83	0.97	Commiphora
320	Commiphora mildbraedii	0.98	0.99	Commiphora
321	Commiphora mollis	0.74	0.91	Commiphora
322	Commiphora mombassensis	0.94	0.96	Commiphora
323	Commiphora monstruosa	0.98	0.99	Commiphora
324	Commiphora mossambicensis	0.84	0.95	Commiphora
325	Commiphora multijuga	0.77	0.96	Commiphora
326	Commiphora myrrha	0.47	0.80	Commiphora
327	Commiphora neglecta	0.89	0.98	Commiphora
328	Commiphora oblanceolata	0.81	0.95	Commiphora
329	Commiphora oblongifolia	0.97	0.99	Commiphora
330	Commiphora orbicularis	0.85	0.95	Commiphora
331	Commiphora ovalifolia	1.00	1.00	Commiphora
332	Commiphora paolii	0.88	0.95	Commiphora
333	Commiphora pedunculata	0.86	0.94	Commiphora
334	Commiphora pervilleana	0.90	0.97	Commiphora
335	Commiphora pteleifolia	0.86	0.97	Commiphora
336	Commiphora pterocarpa	0.73	0.92	Commiphora
337	Commiphora pyracanthoides	0.78	0.92	Commiphora
338	Commiphora quadricincta	0.76	0.93	Commiphora
339	Commiphora rostrata	0.87	0.95	Commiphora
340	Commiphora samharensis	0.72	0.94	Commiphora
341	Commiphora sarandensis	0.79	0.93	Commiphora
342	Commiphora saxicola	0.92	0.97	Commiphora
343	Commiphora schimperi	0.79	0.93	Commiphora
344	Commiphora schlechteri	0.96	1.00	Commiphora
345	Commiphora serrata	0.93	0.96	Commiphora
346	Commiphora serrulata	0.88	0.97	Commiphora
347	Commiphora simplicifolia	0.86	0.95	Commiphora
348	Commiphora sinuata	0.99	0.99	Commiphora
349	Commiphora spathulata	0.74	0.88	Commiphora
350	Commiphora stellulata	0.83	0.88	Commiphora
351	Commiphora swynnertonii	0.88	0.94	Commiphora
352	Commiphora tenuipetiolata	0.77	0.94	Commiphora
353	Commiphora tetramera	0.91	0.96	Commiphora
354	Commiphora truncata	0.78	0.83	Commiphora
355	Commiphora ugogensis	0.90	0.98	Commiphora

	species name	TSS	AUC	group
356	Commiphora viminea	0.81	0.95	Commiphora
357	Commiphora virgata	0.89	0.96	Commiphora
358	Commiphora woodii	0.88	0.97	Commiphora
359	Commiphora zanzibarica	0.86	0.95	Commiphora
360	Copaifera baumiana	0.96	0.98	Detarioideae
361	Cryptosepalum congolanum	0.85	0.96	Detarioideae
362	Cryptosepalum exfoliatum	0.86	0.96	Detarioideae
363	Cryptosepalum katangense	0.96	0.97	Detarioideae
364	Cryptosepalum maraviense	0.89	0.97	Detarioideae
365	Cymbopogon caesius	0.45	0.76	Panicoideae
366	Cymbopogon commutatus	0.53	0.84	Panicoideae
367	Cymbopogon densiflorus	0.82	0.94	Panicoideae
368	Cymbopogon dieterlenii	0.82	0.94	Panicoideae
369	Cymbopogon giganteus	0.68	0.88	Panicoideae
370	Cymbopogon marginatus	0.86	0.96	Panicoideae
371	Cymbopogon nardus	0.62	0.86	Panicoideae
O-372	Cymbopogon nervatus	0.63	0.71	Panicoideae
373	Cymbopogon pospischilii	0.64	0.88	Panicoideae
374	Cymbopogon schoenanthus	0.52	0.79	Panicoideae
375	Cynodon aethiopicus	0.59	0.85	Chloridoideae
376	Cynodon dactylon	0.47	0.80	Chloridoideae
377	Cynodon incompletus	0.78	0.92	Chloridoideae
378	Cynodon nlemfuensis	0.59	0.86	Chloridoideae
379	Cynodon plectostachyus	0.71	0.92	Chloridoideae
380	Cynodon radiatus	0.78	0.91	Chloridoideae
381	Cynodon transvaalensis	0.80	0.94	Chloridoideae
382	Cyrtococcum bosseri	0.84	0.97	Panicoideae
383	Cyrtococcum chaetophoron	0.86	0.95	Panicoideae
384	Cyrtococcum deltoideum	0.88	0.98	Panicoideae
385	Cyrtococcum multinode	0.90	0.93	Panicoideae
386	Cyrtococcum patens	0.85	0.96	Panicoideae
387	Cyrtococcum trigonum	0.86	0.98	Panicoideae
388	Dactyloctenium aegyptium	0.54	0.83	Chloridoideae
389	Dactyloctenium australe	0.81	0.94	Chloridoideae
390	Dactyloctenium capitatum	0.99	1.00	Chloridoideae
391	Dactyloctenium ctenoides	0.77	0.91	Chloridoideae
392	Dactyloctenium geminatum	0.79	0.95	Chloridoideae
393	Dactyloctenium giganteum	0.78	0.94	Chloridoideae
394	Dactyloctenium scindicum	0.65	0.91	Chloridoideae
395	Daniellia alsteeniana	0.86	0.96	Detarioideae
396	Daniellia oliveri	0.76	0.89	Detarioideae
397	Danthoniopsis acutigluma	0.96	0.97	Panicoideae
398	Danthoniopsis barbata	0.76	0.90	Panicoideae
399	Danthoniopsis dinteri	0.74	0.93	Panicoideae
400	Danthoniopsis parva	0.92	0.96	Panicoideae
401	Danthoniopsis pruinosa	0.67	0.85	Panicoideae
402	Danthoniopsis ramosa	0.87	0.95	Panicoideae
403	Danthoniopsis viridis	0.96	0.99	Panicoideae
404	Decaryella madagascariensis	0.92	0.97	Chloridoideae
405	Dichanthium annulatum	0.55	0.78	Panicoideae
406	Dichanthium aristatum	0.68	0.89	Panicoideae
407	Dichanthium foveolatum	0.63	0.84	Panicoideae
408	Digitaria abyssinica	0.65	0.89	Panicoideae
409	Digitaria acuminatissima	0.57	0.85	Panicoideae
410	Digitaria angolensis	0.66	0.87	Panicoideae
411	Digitaria argillacea	0.71	0.88	Panicoideae
412	Digitaria argyrograpta	0.83	0.96	Panicoideae
413	Digitaria argyrotricha	0.81	0.96	Panicoideae
414	Digitaria aridicola	0.81	0.95	Panicoideae
415	Digitaria arushae	0.84	0.94	Panicoideae
416	Digitaria atrofusca	0.80	0.93	Panicoideae
O-417	Digitaria barbinodis	0.55	0.70	Panicoideae
418	Digitaria bicornis	0.64	0.87	Panicoideae
419	Digitaria brazzae	0.75	0.94	Panicoideae
420	Digitaria ciliaris	0.49	0.82	Panicoideae
421	Digitaria comifera	0.76	0.85	Panicoideae
422	Digitaria compressa	0.79	0.89	Panicoideae
423	Digitaria debilis	0.60	0.84	Panicoideae
424	Digitaria delicata	0.75	0.86	Panicoideae
425	Digitaria delicatula	0.87	0.95	Panicoideae
426	Digitaria diagonalis	0.70	0.90	Panicoideae
427	Digitaria didactyla	0.76	0.91	Panicoideae

	species name	TSS	AUC	group
428	Digitaria diversinervis	0.90	0.98	Panicoideae
429	Digitaria eriantha	0.60	0.84	Panicoideae
430	Digitaria exilis	0.65	0.86	Panicoideae
431	Digitaria eylesii	0.84	0.94	Panicoideae
432	Digitaria flaccida	0.88	0.94	Panicoideae
433	Digitaria fuscescens	0.81	0.95	Panicoideae
434	Digitaria gayana	0.58	0.84	Panicoideae
435	Digitaria gazensis	0.83	0.94	Panicoideae
436	Digitaria glauca	0.85	0.87	Panicoideae
437	Digitaria gymnostachys	0.87	0.96	Panicoideae
438	Digitaria horizontalis	0.61	0.88	Panicoideae
439	Digitaria humbertii	0.91	0.97	Panicoideae
440	Digitaria hyalina	0.97	0.98	Panicoideae
441	Digitaria leptorhachis	0.80	0.93	Panicoideae
442	Digitaria longiflora	0.62	0.87	Panicoideae
443	Digitaria macroblephara	0.77	0.95	Panicoideae
444	Digitaria maitlandii	0.88	0.97	Panicoideae
445	Digitaria maniculata	0.72	0.88	Panicoideae
446	Digitaria milanjiana	0.76	0.91	Panicoideae
447	Digitaria monodactyla	0.81	0.94	Panicoideae
448	Digitaria natalensis	0.80	0.96	Panicoideae
449	Digitaria nuda	0.60	0.86	Panicoideae
450	Digitaria pearsonii	0.71	0.91	Panicoideae
451	Digitaria pennata	0.68	0.91	Panicoideae
452	Digitaria perrottetii	0.65	0.90	Panicoideae
453	Digitaria poggeana	0.88	0.95	Panicoideae
454	Digitaria polyphylla	0.96	0.99	Panicoideae
455	Digitaria pseudodiagonalis	0.82	0.91	Panicoideae
456	Digitaria radicosa	0.78	0.93	Panicoideae
457	Digitaria remotigluma	0.83	0.91	Panicoideae
458	Digitaria rivae	0.79	0.94	Panicoideae
459	Digitaria sanguinalis	0.57	0.84	Panicoideae
460	Digitaria schmitzii	0.84	0.91	Panicoideae
461	Digitaria seriata	0.87	0.96	Panicoideae
462	Digitaria setifolia	0.86	0.96	Panicoideae
463	Digitaria siderograpta	0.77	0.94	Panicoideae
464	Digitaria ternata	0.70	0.89	Panicoideae
465	Digitaria thouarsiana	0.70	0.86	Panicoideae
466	Digitaria tisserantii	0.96	0.98	Panicoideae
467	Digitaria tricholaenoides	0.91	0.98	Panicoideae
468	Digitaria velutina	0.60	0.87	Panicoideae
469	Digitaria violascens	0.67	0.89	Panicoideae
470	Dignathia gracilis	0.70	0.90	Chloridoideae
471	Dignathia hirtella	0.69	0.92	Chloridoideae
472	Dignathia villosa	0.92	0.95	Chloridoideae
473	Diheteropogon amplectens	0.61	0.84	Panicoideae
474	Diheteropogon filifolius	0.77	0.93	Panicoideae
475	Diheteropogon hagerupii	0.83	0.95	Panicoideae
476	Dilophotriche occidentalis	0.91	0.92	Panicoideae
477	Dilophotriche pobeguinii	0.85	0.95	Panicoideae
478	Dilophotriche tristachyoides	0.85	0.92	Panicoideae
479	Dinebra haareri	0.79	0.93	Chloridoideae
480	Dinebra perrieri	0.88	0.94	Chloridoideae
481	Dinebra polycarpha	0.98	0.99	Chloridoideae
482	Dinebra retroflexa	0.46	0.77	Chloridoideae
483	Eccoptocarpha obconiciventris	0.89	0.91	Panicoideae
484	Echinochloa brevipedicellata	0.86	0.92	Panicoideae
485	Echinochloa callopus	0.77	0.88	Panicoideae
486	Echinochloa colonum	0.47	0.80	Panicoideae
487	Echinochloa crus-galli	0.53	0.81	Panicoideae
488	Echinochloa crus-pavonis	0.57	0.86	Panicoideae
489	Echinochloa frumentacea	0.66	0.89	Panicoideae
490	Echinochloa haploclada	0.82	0.95	Panicoideae
491	Echinochloa jubata	0.76	0.89	Panicoideae
492	Echinochloa obtusiflora	0.83	0.94	Panicoideae
493	Echinochloa pyramidalis	0.52	0.81	Panicoideae
494	Echinochloa rotundiflora	0.85	0.95	Panicoideae
495	Echinochloa stagnina	0.55	0.79	Panicoideae
496	Echinochloa ugandensis	0.75	0.91	Panicoideae
497	Eleusine africana	0.47	0.78	Chloridoideae
498	Eleusine coracana	0.53	0.83	Chloridoideae
499	Eleusine floccifolia	0.86	0.92	Chloridoideae

	species name	TSS	AUC	group
500	Eleusine indica	0.55	0.85	Chloridoideae
501	Eleusine intermedia	0.89	0.95	Chloridoideae
502	Eleusine jaegeri	0.83	0.95	Chloridoideae
503	Eleusine multiflora	0.80	0.95	Chloridoideae
504	Elionurus ciliaris	0.73	0.89	Panicoideae
505	Elionurus elegans	0.79	0.93	Panicoideae
506	Elionurus euchaetus	0.92	0.95	Panicoideae
507	Elionurus hirtifolius	0.83	0.95	Panicoideae
508	Elionurus muticus	0.62	0.85	Panicoideae
509	Elionurus platypus	0.79	0.95	Panicoideae
510	Elionurus royleanus	0.83	0.89	Panicoideae
511	Elionurus tripsacoides	0.74	0.89	Panicoideae
512	Elymandra androphila	0.81	0.94	Panicoideae
513	Elymandra archaelymandra	0.80	0.91	Panicoideae
514	Elymandra gossweileri	0.85	0.97	Panicoideae
515	Elymandra grallata	0.79	0.90	Panicoideae
516	Enneapogon cenchroides	0.64	0.86	Chloridoideae
517	Enneapogon desvauxii	0.49	0.78	Chloridoideae
518	Enneapogon persicus	0.50	0.78	Chloridoideae
519	Enneapogon pretoriensis	0.94	0.97	Chloridoideae
520	Enneapogon scaber	0.69	0.90	Chloridoideae
521	Enneapogon scoparius	0.68	0.87	Chloridoideae
522	Enteropogon macrostachyus	0.66	0.88	Chloridoideae
523	Enteropogon monostachyos	0.71	0.86	Chloridoideae
524	Enteropogon prieurii	0.72	0.90	Chloridoideae
525	Enteropogon rupestris	0.74	0.90	Chloridoideae
526	Enteropogon sechellensis	0.76	0.88	Chloridoideae
527	Entolasia imbricata	0.84	0.96	Panicoideae
528	Entolasia olivacea	0.89	0.97	Panicoideae
529	Entoplocamia aristulata	0.91	0.98	Chloridoideae
530	Eragrostiella bifaria	0.68	0.87	Chloridoideae
531	Eragrostis acraea	0.83	0.95	Chloridoideae
532	Eragrostis aegyptiaca	0.64	0.85	Chloridoideae
533	Eragrostis aethiopica	0.68	0.89	Chloridoideae
534	Eragrostis ambleia	0.95	0.98	Chloridoideae
535	Eragrostis anacrantha	1.00	1.00	Chloridoideae
536	Eragrostis annulata	0.83	0.96	Chloridoideae
537	Eragrostis arenicola	0.70	0.88	Chloridoideae
538	Eragrostis aspera	0.62	0.84	Chloridoideae
539	Eragrostis atrovirens	0.59	0.85	Chloridoideae
540	Eragrostis barbinodis	0.84	0.96	Chloridoideae
541	Eragrostis barrelieri	0.58	0.86	Chloridoideae
542	Eragrostis barteri	0.71	0.88	Chloridoideae
543	Eragrostis bergiana	0.90	0.99	Chloridoideae
544	Eragrostis bicolor	0.90	0.98	Chloridoideae
545	Eragrostis biflora	0.86	0.96	Chloridoideae
546	Eragrostis blepharostachya	0.91	0.97	Chloridoideae
547	Eragrostis boinensis	0.83	0.93	Chloridoideae
548	Eragrostis botryodes	0.89	0.96	Chloridoideae
549	Eragrostis brainii	0.84	0.97	Chloridoideae
550	Eragrostis braunii	0.78	0.92	Chloridoideae
551	Eragrostis brizantha	0.87	0.97	Chloridoideae
552	Eragrostis caesia	0.85	0.95	Chloridoideae
553	Eragrostis caespitosa	0.72	0.89	Chloridoideae
554	Eragrostis canescens	0.97	0.99	Chloridoideae
555	Eragrostis caniflora	0.83	0.92	Chloridoideae
556	Eragrostis capensis	0.71	0.92	Chloridoideae
557	Eragrostis capuronii	0.92	0.96	Chloridoideae
558	Eragrostis castellaneana	0.91	0.96	Chloridoideae
559	Eragrostis cenolepis	0.79	0.90	Chloridoideae
560	Eragrostis chabouisii	1.00	1.00	Chloridoideae
561	Eragrostis chalarothyrsos	0.78	0.91	Chloridoideae
562	Eragrostis chapelieri	0.75	0.91	Chloridoideae
563	Eragrostis cilianensis	0.46	0.78	Chloridoideae
564	Eragrostis ciliaris	0.61	0.86	Chloridoideae
565	Eragrostis cimicina	0.95	0.97	Chloridoideae
566	Eragrostis congesta	0.87	0.94	Chloridoideae
567	Eragrostis crassinervis	0.58	0.82	Chloridoideae
568	Eragrostis curvula	0.65	0.87	Chloridoideae
569	Eragrostis cylindriflora	0.71	0.90	Chloridoideae
570	Eragrostis dinteri	0.74	0.87	Chloridoideae
571	Eragrostis echinochloidea	0.82	0.94	Chloridoideae

	species name	TSS	AUC	group
572	Eragrostis egregia	0.89	0.95	Chloridoideae
573	Eragrostis elegantissima	0.77	0.88	Chloridoideae
574	Eragrostis exasperata	0.79	0.94	Chloridoideae
575	Eragrostis flavicans	0.96	0.96	Chloridoideae
576	Eragrostis gangetica	0.55	0.83	Chloridoideae
577	Eragrostis glandulosipedata	0.73	0.86	Chloridoideae
578	Eragrostis gummiflua	0.85	0.96	Chloridoideae
579	Eragrostis habrantha	0.91	0.97	Chloridoideae
580	Eragrostis heteromera	0.75	0.93	Chloridoideae
581	Eragrostis hierniana	0.88	0.96	Chloridoideae
582	Eragrostis hispida	0.85	0.93	Chloridoideae
583	Eragrostis homomalla	0.85	0.96	Chloridoideae
584	Eragrostis humbertii	1.00	1.00	Chloridoideae
585	Eragrostis humidicola	0.87	0.95	Chloridoideae
586	Eragrostis inamoena	0.81	0.93	Chloridoideae
587	Eragrostis invalida	0.89	0.98	Chloridoideae
588	Eragrostis japonica	0.55	0.82	Chloridoideae
589	Eragrostis laevissima	0.90	0.97	Chloridoideae
590	Eragrostis lappula	0.83	0.95	Chloridoideae
591	Eragrostis lateritica	0.95	0.99	Chloridoideae
592	Eragrostis lehmanniana	0.74	0.91	Chloridoideae
593	Eragrostis lingulata	0.84	0.94	Chloridoideae
594	Eragrostis macilenta	0.78	0.90	Chloridoideae
595	Eragrostis macrochlamys	0.84	0.94	Chloridoideae
596	Eragrostis membranacea	0.94	0.97	Chloridoideae
597	Eragrostis micrantha	0.85	0.98	Chloridoideae
598	Eragrostis mildbraedii	0.82	0.95	Chloridoideae
599	Eragrostis minor	0.59	0.83	Chloridoideae
600	Eragrostis moggii	0.94	1.00	Chloridoideae
601	Eragrostis mollior	0.95	0.99	Chloridoideae
602	Eragrostis muerensis	0.96	0.97	Chloridoideae
603	Eragrostis nindensis	0.73	0.92	Chloridoideae
604	Eragrostis obtusa	0.85	0.96	Chloridoideae
605	Eragrostis olivacea	0.76	0.91	Chloridoideae
606	Eragrostis omahekensis	0.91	0.97	Chloridoideae
607	Eragrostis pallens	0.85	0.96	Chloridoideae
608	Eragrostis paniciformis	0.79	0.90	Chloridoideae
609	Eragrostis papposa	0.65	0.87	Chloridoideae
610	Eragrostis paradoxa	0.96	0.98	Chloridoideae
611	Eragrostis patens	0.79	0.94	Chloridoideae
612	Eragrostis patentipilosa	0.87	0.97	Chloridoideae
613	Eragrostis patentissima	0.90	0.97	Chloridoideae
614	Eragrostis patula	0.79	0.94	Chloridoideae
615	Eragrostis perbella	0.86	0.96	Chloridoideae
616	Eragrostis phyllacantha	0.93	0.97	Chloridoideae
617	Eragrostis pilgeriana	0.94	0.98	Chloridoideae
618	Eragrostis pilosa	0.41	0.75	Chloridoideae
619	Eragrostis plana	0.78	0.92	Chloridoideae
620	Eragrostis planiculmis	0.85	0.96	Chloridoideae
621	Eragrostis plurigluma	0.85	0.96	Chloridoideae
622	Eragrostis porosa	0.81	0.95	Chloridoideae
623	Eragrostis procumbens	0.88	0.97	Chloridoideae
624	Eragrostis prolifera	0.73	0.91	Chloridoideae
625	Eragrostis pycnostachys	0.93	0.99	Chloridoideae
626	Eragrostis racemosa	0.74	0.90	Chloridoideae
627	Eragrostis remotiflora	0.90	0.97	Chloridoideae
628	Eragrostis rogersii	0.88	0.94	Chloridoideae
629	Eragrostis rotifer	0.78	0.93	Chloridoideae
630	Eragrostis sabinae	0.75	0.81	Chloridoideae
631	Eragrostis sarmentosa	0.76	0.90	Chloridoideae
632	Eragrostis schweinfurthii	0.83	0.95	Chloridoideae
633	Eragrostis sclerantha	0.86	0.96	Chloridoideae
634	Eragrostis setulifera	0.70	0.91	Chloridoideae
635	Eragrostis soratensis	0.72	0.84	Chloridoideae
636	Eragrostis squamata	0.70	0.87	Chloridoideae
637	Eragrostis stapfii	0.87	0.98	Chloridoideae
638	Eragrostis subglandulosa	0.94	0.95	Chloridoideae
639	Eragrostis superba	0.56	0.83	Chloridoideae
640	Eragrostis tef	0.65	0.88	Chloridoideae
641	Eragrostis tenella	0.63	0.87	Chloridoideae
642	Eragrostis tenuifolia	0.64	0.88	Chloridoideae
643	Eragrostis thollonii	0.87	0.96	Chloridoideae

	species name	TSS	AUC	group
644	Eragrostis tremula	0.53	0.81	Chloridoideae
645	Eragrostis trimucronata	0.96	0.97	Chloridoideae
646	Eragrostis truncata	0.94	0.98	Chloridoideae
647	Eragrostis turgida	0.78	0.92	Chloridoideae
648	Eragrostis unioloides	0.77	0.92	Chloridoideae
649	Eragrostis virescens	0.62	0.85	Chloridoideae
650	Eragrostis viscosa	0.64	0.87	Chloridoideae
651	Eragrostis volkensii	0.86	0.95	Chloridoideae
652	Eragrostis welwitschii	0.78	0.94	Chloridoideae
653	Eriochloa macclounii	0.88	0.93	Panicoideae
654	Eriochloa meyeriana	0.70	0.93	Panicoideae
655	Eriochloa parvispiculata	0.76	0.93	Panicoideae
656	Eriochloa procera	0.64	0.85	Panicoideae
657	Eriochloa rovumensis	0.89	0.91	Panicoideae
658	Eriochloa stapfiana	0.90	0.96	Panicoideae
659	Eriochloa subulifera	0.78	0.85	Panicoideae
660	Eriochrysis brachypogon	0.72	0.90	Panicoideae
661	Eriochrysis pallida	0.78	0.93	Panicoideae
662	Eriochrysis purpurata	0.95	0.99	Panicoideae
663	Euclasta condylotricha	0.74	0.89	Panicoideae
664	Eulalia aurea	0.71	0.89	Panicoideae
665	Eulalia polyneura	0.98	0.99	Panicoideae
666	Eulalia villosa	0.82	0.96	Panicoideae
667	Exotheca abyssinica	0.79	0.93	Panicoideae
668	Fingerhuthia africana	0.78	0.93	Chloridoideae
669	Fingerhuthia sesleriiformis	0.84	0.96	Chloridoideae
670	Gilgiochloa indurata	0.89	0.95	Panicoideae
671	Gilletiodendron glandulosum	0.93	0.97	Detarioideae
672	Guibourtia carrissoana	0.90	0.96	Detarioideae
673	Guibourtia coleosperma	0.85	0.96	Detarioideae
674	Guibourtia conjugata	0.90	0.96	Detarioideae
675	Guibourtia copallifera	0.78	0.92	Detarioideae
676	Guibourtia schliebenii	0.85	0.95	Detarioideae
677	Guiera senegalensis	0.79	0.92	Combretaceae
678	Habrochloa bullockii	0.93	0.94	Chloridoideae
679	Hackelochloa granularis	0.66	0.86	Panicoideae
680	Hemarthria altissima	0.54	0.82	Panicoideae
681	Hemarthria natans	0.55	0.88	Panicoideae
682	Heteropholis sulcata	0.92	0.95	Panicoideae
683	Heteropogon contortus	0.54	0.83	Panicoideae
684	Heteropogon melanocarpus	0.62	0.86	Panicoideae
685	Homozeugos eylesii	0.97	0.97	Panicoideae
686	Hydrothauma manicatum	0.92	0.97	Panicoideae
687	Hylebates chlorochloe	0.89	0.99	Panicoideae
688	Hylebates cordatus	0.89	0.93	Panicoideae
689	Hyparrhenia anamesa	0.81	0.94	Panicoideae
690	Hyparrhenia anthistirioides	0.82	0.91	Panicoideae
691	Hyparrhenia bagirmica	0.86	0.96	Panicoideae
692	Hyparrhenia barteri	0.77	0.90	Panicoideae
693	Hyparrhenia bracteata	0.78	0.94	Panicoideae
694	Hyparrhenia collina	0.88	0.95	Panicoideae
695	Hyparrhenia confinis	0.87	0.96	Panicoideae
696	Hyparrhenia cyanescens	0.73	0.91	Panicoideae
697	Hyparrhenia cymbaria	0.78	0.94	Panicoideae
698	Hyparrhenia dichroa	0.77	0.92	Panicoideae
699	Hyparrhenia diplandra	0.72	0.91	Panicoideae
700	Hyparrhenia dregeana	0.89	0.96	Panicoideae
701	Hyparrhenia exarmata	0.73	0.88	Panicoideae
702	Hyparrhenia familiaris	0.82	0.95	Panicoideae
703	Hyparrhenia figariana	0.79	0.88	Panicoideae
704	Hyparrhenia filipendula	0.72	0.91	Panicoideae
705	Hyparrhenia finitima	0.62	0.85	Panicoideae
706	Hyparrhenia formosa	0.88	0.98	Panicoideae
707	Hyparrhenia gazensis	0.84	0.95	Panicoideae
708	Hyparrhenia glabriuscula	0.88	0.96	Panicoideae
709	Hyparrhenia gossweileri	0.99	0.99	Panicoideae
710	Hyparrhenia hirta	0.63	0.86	Panicoideae
711	Hyparrhenia involucrata	0.81	0.94	Panicoideae
712	Hyparrhenia madaropoda	0.72	0.91	Panicoideae
713	Hyparrhenia multiplex	0.88	0.94	Panicoideae
714	Hyparrhenia newtonii	0.79	0.95	Panicoideae
715	Hyparrhenia niariensis	0.83	0.95	Panicoideae

	species name	TSS	AUC	group
716	Hyparrhenia nyassae	0.77	0.91	Panicoideae
717	Hyparrhenia papillipes	0.80	0.94	Panicoideae
718	Hyparrhenia pilgeriana	0.76	0.92	Panicoideae
719	Hyparrhenia poecilotricha	0.80	0.93	Panicoideae
720	Hyparrhenia quarrei	0.74	0.89	Panicoideae
721	Hyparrhenia rudis	0.67	0.85	Panicoideae
722	Hyparrhenia rufa	0.66	0.89	Panicoideae
723	Hyparrhenia schimperi	0.79	0.93	Panicoideae
724	Hyparrhenia smithiana	0.78	0.91	Panicoideae
725	Hyparrhenia subplumosa	0.77	0.91	Panicoideae
726	Hyparrhenia tamba	0.82	0.96	Panicoideae
727	Hyparrhenia umbrosa	0.81	0.96	Panicoideae
728	Hyparrhenia variabilis	0.77	0.91	Panicoideae
729	Hyparrhenia violascens	0.87	0.95	Panicoideae
730	Hyparrhenia welwitschii	0.77	0.94	Panicoideae
731	Hyperthelia cornucopiae	0.89	0.97	Panicoideae
732	Hyperthelia dissoluta	0.63	0.85	Panicoideae
733	Icuria dunensis	1.00	1.00	Detarioideae
734	Imperata cylindrica	0.52	0.82	Panicoideae
735	Intsia bijuga	0.77	0.95	Detarioideae
736	Ischaemum afrum	0.61	0.88	Panicoideae
737	Ischaemum amethystinum	0.84	0.95	Panicoideae
738	Ischaemum koleostachys	0.75	0.93	Panicoideae
739	Ischaemum polystachyum	0.72	0.92	Panicoideae
740	Ischaemum rugosum	0.70	0.89	Panicoideae
741	Isoberlinia angolensis	0.84	0.95	Detarioideae
742	Isoberlinia doka	0.81	0.92	Detarioideae
743	Isoberlinia tomentosa	0.77	0.93	Detarioideae
744	Julbernardia globiflora	0.81	0.94	Detarioideae
745	Julbernardia paniculata	0.82	0.96	Detarioideae
746	Julbernardia unijugata	0.98	0.99	Detarioideae
747	Lasiurus scindicus	0.71	0.91	Panicoideae
748	Leptocarydion vulpiastrum	0.75	0.93	Chloridoideae
749	Leptothrium senegalense	0.61	0.89	Chloridoideae
750	Lepturus anadabolavensis	0.87	0.96	Chloridoideae
751	Lepturus boinensis	0.90	0.91	Chloridoideae
752	Lepturus humbertianus	0.71	0.88	Chloridoideae
753	Lepturus radicans	0.80	0.94	Chloridoideae
754	Lepturus repens	0.88	0.97	Chloridoideae
755	Lophacme digitata	0.87	0.95	Chloridoideae
756	Loudetia annua	0.82	0.94	Panicoideae
757	Loudetia arundinacea	0.77	0.91	Panicoideae
758	Loudetia coarctata	0.89	0.95	Panicoideae
759	Loudetia densispica	0.91	0.98	Panicoideae
760	Loudetia filifolia	0.82	0.93	Panicoideae
761	Loudetia flavida	0.66	0.86	Panicoideae
762	Loudetia hordeiformis	0.84	0.93	Panicoideae
763	Loudetia kagerensis	0.75	0.93	Panicoideae
764	Loudetia lanata	0.95	0.98	Panicoideae
765	Loudetia phragmitoides	0.79	0.94	Panicoideae
766	Loudetia simplex	0.68	0.87	Panicoideae
767	Loudetia togoensis	0.80	0.92	Panicoideae
768	Loudetia vanderystii	0.90	0.97	Panicoideae
769	Loudetiopsis ambiens	0.84	0.96	Panicoideae
770	Loudetiopsis capillipes	0.90	0.93	Panicoideae
771	Loudetiopsis chrysothrix	0.80	0.93	Panicoideae
772	Loudetiopsis glabrata	0.84	0.94	Panicoideae
773	Loudetiopsis kerstingii	0.86	0.95	Panicoideae
774	Loudetiopsis scaettae	0.89	0.97	Panicoideae
775	Loudetiopsis thoroldii	0.84	0.92	Panicoideae
776	Loudetiopsis trigemina	0.85	0.95	Panicoideae
777	Louisiella fluitans	0.73	0.88	Panicoideae
778	Loxodera ledermannii	0.86	0.95	Panicoideae
779	Megastachya mucronata	0.81	0.95	Panicoideae
O-780	Melanocenchris abyssinica	0.45	0.65	Chloridoideae
781	Melinis ambigua	0.92	0.96	Panicoideae
782	Melinis amethystea	0.86	0.95	Panicoideae
783	Melinis effusa	0.73	0.91	Panicoideae
784	Melinis kallimorpha	0.97	0.99	Panicoideae
785	Melinis longiseta	0.73	0.93	Panicoideae
786	Melinis macrochaeta	0.75	0.92	Panicoideae
787	Melinis minutiflora	0.72	0.92	Panicoideae

	species name	TSS	AUC	group
788	Melinis nerviglumis	0.68	0.90	Panicoideae
789	Melinis repens	0.60	0.85	Panicoideae
790	Melinis scabrida	0.93	0.98	Panicoideae
791	Melinis subglabra	0.77	0.87	Panicoideae
792	Melinis tenuissima	0.85	0.95	Panicoideae
793	Mesosetum rottboellioides	0.76	0.95	Panicoideae
794	Micrachne obtusiflora	0.72	0.83	Chloridoideae
795	Miscanthus ecklonii	0.85	0.95	Panicoideae
796	Mnesithea laevis	0.69	0.89	Panicoideae
797	Monelytrum luederitzianum	0.89	0.95	Chloridoideae
798	Monocymbium ceresiiforme	0.65	0.87	Panicoideae
799	Monocymbium deightonii	0.90	0.97	Panicoideae
800	Mosdenia leptostachys	0.92	0.99	Chloridoideae
801	Neoapaloxylon madagascariense	0.84	0.95	Detarioideae
802	Neoapaloxylon mandrarense	0.65	0.88	Detarioideae
803	Neoapaloxylon tuberosum	0.82	0.92	Detarioideae
804	Neostapfiella chloridiantha	0.88	0.92	Chloridoideae
805	Neostapfiella perrieri	0.85	0.93	Chloridoideae
806	Neyraudia arundinacea	0.76	0.91	Chloridoideae
807	Odyssea paucinervis	0.80	0.94	Chloridoideae
808	Ophiuros papillosus	0.95	0.99	Panicoideae
809	Oplismenus burmanni	0.69	0.89	Panicoideae
810	Oplismenus compositus	0.71	0.92	Panicoideae
811	Oplismenus flavicomus	0.82	0.93	Panicoideae
812	Oplismenus hirtellus	0.70	0.91	Panicoideae
813	Oplismenus undulatifolius	0.76	0.94	Panicoideae
814	Oropetium aristatum	0.88	0.95	Chloridoideae
815	Oropetium capense	0.79	0.93	Chloridoideae
816	Oropetium thomaeum	0.80	0.94	Chloridoideae
817	Orthoclada africana	0.87	0.91	Panicoideae
818	Oryzidium barnardii	0.92	0.96	Panicoideae
819	Oxyrhachis gracillima	0.86	0.95	Panicoideae
820	Panicum acrotrichum	0.84	0.93	Panicoideae
821	Panicum aequinerve	0.90	0.97	Panicoideae
822	Panicum afzelii	0.76	0.93	Panicoideae
823	Panicum ambositrense	0.95	0.97	Panicoideae
824	Panicum anabaptistum	0.80	0.93	Panicoideae
825	Panicum andringitrense	0.88	0.96	Panicoideae
826	Panicum antidotale	0.60	0.83	Panicoideae
827	Panicum arcurameum	0.89	0.97	Panicoideae
828	Panicum atrosanguineum	0.56	0.84	Panicoideae
829	Panicum brevifolium	0.74	0.93	Panicoideae
830	Panicum callosum	0.84	0.90	Panicoideae
831	Panicum calvum	0.81	0.96	Panicoideae
832	Panicum chionachne	0.86	0.94	Panicoideae
833	Panicum cinctum	0.88	0.96	Panicoideae
834	Panicum coloratum	0.51	0.81	Panicoideae
835	Panicum comorense	0.76	0.92	Panicoideae
836	Panicum congoense	0.83	0.95	Panicoideae
837	Panicum delicatulum	0.96	0.98	Panicoideae
838	Panicum deustum	0.71	0.91	Panicoideae
839	Panicum dewinteri	1.00	1.00	Panicoideae
840	Panicum dregeanum	0.73	0.91	Panicoideae
841	Panicum fluviicola	0.68	0.88	Panicoideae
842	Panicum genuflexum	0.76	0.94	Panicoideae
843	Panicum gilvum	0.77	0.92	Panicoideae
844	Panicum glandulopaniculatum	0.86	0.94	Panicoideae
845	Panicum griffonii	0.83	0.96	Panicoideae
846	Panicum haplocaulos	0.92	0.98	Panicoideae
847	Panicum hirtum	0.74	0.91	Panicoideae
848	Panicum hochstetteri	0.66	0.91	Panicoideae
849	Panicum homblei	0.90	0.94	Panicoideae
850	Panicum humile	0.67	0.83	Panicoideae
851	Panicum hygrocharis	0.69	0.87	Panicoideae
852	Panicum ibitense	0.89	0.95	Panicoideae
853	Panicum impeditum	0.85	0.97	Panicoideae
854	Panicum inaequilatum	0.96	0.98	Panicoideae
855	Panicum infestum	0.78	0.94	Panicoideae
856	Panicum issongense	0.88	0.93	Panicoideae
857	Panicum kalaharense	0.89	0.97	Panicoideae
858	Panicum laetum	0.70	0.88	Panicoideae
859	Panicum lanipes	0.95	0.99	Panicoideae

	species name	TSS	AUC	group
860	Panicum laticomum	0.82	0.92	Panicoideae
861	Panicum luridum	0.77	0.87	Panicoideae
862	Panicum madipirense	0.89	0.91	Panicoideae
863	Panicum malacotrichum	0.86	0.97	Panicoideae
864	Panicum massaiense	0.79	0.90	Panicoideae
865	Panicum maximum	0.65	0.89	Panicoideae
866	Panicum merkeri	0.85	0.92	Panicoideae
867	Panicum miliaceum	0.64	0.87	Panicoideae
868	Panicum mitopus	0.93	0.96	Panicoideae
869	Panicum mlahiense	0.93	0.93	Panicoideae
870	Panicum monticola	0.84	0.97	Panicoideae
871	Panicum nigerense	0.75	0.90	Panicoideae
872	Panicum novemnerve	0.80	0.94	Panicoideae
873	Panicum nudiflorum	0.95	0.97	Panicoideae
874	Panicum pansum	0.69	0.87	Panicoideae
875	Panicum paucinode	0.86	0.96	Panicoideae
876	Panicum perrieri	0.95	1.00	Panicoideae
877	Panicum phragmitoides	0.72	0.90	Panicoideae
878	Panicum pilgeri	0.84	0.95	Panicoideae
879	Panicum pilgerianum	0.98	1.00	Panicoideae
880	Panicum pleianthum	0.85	0.97	Panicoideae
881	Panicum porphyrrhizos	0.76	0.89	Panicoideae
882	Panicum pusillum	0.84	0.94	Panicoideae
883	Panicum repens	0.56	0.84	Panicoideae
884	Panicum schinzii	0.66	0.89	Panicoideae
885	Panicum simulans	0.91	0.97	Panicoideae
886	Panicum spongiosum	0.70	0.87	Panicoideae
887	Panicum stapfianum	0.82	0.96	Panicoideae
888	Panicum subalbidum	0.51	0.76	Panicoideae
889	Panicum subhystrix	0.90	0.98	Panicoideae
890	Panicum sumatrense	0.69	0.83	Panicoideae
891	Panicum trichocladum	0.78	0.93	Panicoideae
892	Panicum trichoides	0.73	0.91	Panicoideae
893	Panicum trichonode	0.99	0.99	Panicoideae
894	Panicum turgidum	0.59	0.83	Panicoideae
895	Panicum voeltzkowii	0.78	0.90	Panicoideae
896	Panicum volutans	0.90	0.98	Panicoideae
897	Parahyparrhenia annua	0.84	0.94	Panicoideae
898	Paratheria prostrata	0.71	0.90	Panicoideae
899	Paspalum conjugatum	0.75	0.93	Panicoideae
900	Paspalum dilatatum	0.62	0.86	Panicoideae
901	Paspalum distichum	0.58	0.86	Panicoideae
902	Paspalum glumaceum	0.87	0.94	Panicoideae
903	Paspalum lamprocaryon	0.75	0.91	Panicoideae
904	Paspalum notatum	0.69	0.90	Panicoideae
905	Paspalum paniculatum	0.76	0.93	Panicoideae
906	Paspalum scrobiculatum	0.62	0.88	Panicoideae
907	Paspalum urvillei	0.70	0.91	Panicoideae
908	Paspalum vaginatum	0.56	0.86	Panicoideae
909	Paspalum virgatum	0.74	0.93	Panicoideae
910	Perotis flavinodula	1.00	1.00	Chloridoideae
911	Perotis hildebrandtii	0.80	0.91	Chloridoideae
912	Perotis indica	0.73	0.92	Chloridoideae
913	Perotis leptopus	0.87	0.95	Chloridoideae
914	Perotis patens	0.68	0.89	Chloridoideae
915	Perotis scabra	0.79	0.90	Chloridoideae
916	Perotis vaginata	0.80	0.91	Chloridoideae
917	Phacelurus gabonensis	0.77	0.93	Panicoideae
918	Poecilostachys bakeri	0.93	0.97	Panicoideae
919	Poecilostachys baronis	0.94	0.99	Panicoideae
920	Poecilostachys hildebrandtii	0.86	0.94	Panicoideae
921	Poecilostachys humbertii	0.75	0.90	Panicoideae
922	Poecilostachys oplismenoides	0.86	0.97	Panicoideae
923	Prioria msoo	0.71	0.89	Detarioideae
924	Pseudechinolaena polystachya	0.81	0.95	Panicoideae
925	Rhytachne gracilis	0.85	0.92	Panicoideae
926	Rhytachne latifolia	0.84	0.96	Panicoideae
927	Rhytachne rottboellioides	0.75	0.91	Panicoideae
928	Rhytachne triaristata	0.81	0.93	Panicoideae
929	Rottboellia cochinchinensis	0.64	0.88	Panicoideae
930	Rottboellia purpurascens	0.79	0.89	Panicoideae
931	Sartidia dewinteri	0.98	1.00	Aristidoideae

	species name	TSS	AUC	group
932	Sartidia isaloensis	0.96	0.97	Aristidoideae
933	Sartidia jucunda	0.94	0.98	Aristidoideae
934	Sartidia vanderystii	0.80	0.89	Aristidoideae
935	Schizachyrium brevifolium	0.66	0.89	Panicoideae
936	Schizachyrium claudopus	1.00	1.00	Panicoideae
937	Schizachyrium delicatum	0.86	0.93	Panicoideae
938	Schizachyrium exile	0.62	0.84	Panicoideae
939	Schizachyrium gresicola	0.72	0.91	Panicoideae
940	Schizachyrium jeffreysii	0.85	0.96	Panicoideae
941	Schizachyrium maclaudii	0.81	0.95	Panicoideae
942	Schizachyrium nodulosum	0.81	0.94	Panicoideae
943	Schizachyrium platyphyllum	0.75	0.92	Panicoideae
944	Schizachyrium pulchellum	0.91	0.97	Panicoideae
945	Schizachyrium ruderale	0.83	0.94	Panicoideae
946	Schizachyrium sanguineum	0.60	0.86	Panicoideae
947	Schizachyrium scintillans	0.85	0.95	Panicoideae
948	Schizachyrium thollonii	0.85	0.98	Panicoideae
949	Schizachyrium urceolatum	0.87	0.97	Panicoideae
950	Schmidtia kalahariensis	0.79	0.91	Panicoideae
951	Schmidtia pappophoroides	0.65	0.86	Chloridoideae
952	Schotia afra	0.87	0.97	Detarioideae
953	Schotia brachypetala	0.80	0.95	Detarioideae
954	Schotia capitata	0.89	0.98	Detarioideae
955	Schotia latifolia	0.86	0.96	Detarioideae
956	Sclerodactylon macrostachyum	0.81	0.93	Chloridoideae
957	Sehima galpinii	0.94	0.99	Panicoideae
958	Sehima ischaemoides	0.50	0.76	Panicoideae
O-959	Senegalia adenocalyx	0.84	0.93	Acacia - not included
O-960	Senegalia asak	0.60	0.84	Acacia - not included
O-961	Senegalia ataxacantha	0.56	0.83	Acacia - not included
O-962	Senegalia brevispica	0.68	0.91	Acacia - not included
O-963	Senegalia burkei	0.89	0.98	Acacia - not included
O-964	Senegalia caffra	0.78	0.94	Acacia - not included
O-965	Senegalia galpinii	0.72	0.90	Acacia - not included
O-966	Senegalia goetzei	0.75	0.91	Acacia - not included
O-967	Senegalia hecatophylla	0.71	0.88	Acacia - not included
O-968	Senegalia hereroensis	0.84	0.93	Acacia - not included
O-969	Senegalia hildebrandtii	0.75	0.96	Acacia - not included
O-970	Senegalia kraussiana	0.77	0.95	Acacia - not included
O-971	Senegalia laeta	0.58	0.80	Acacia - not included
O-972	Senegalia macrostachya	0.77	0.88	Acacia - not included
O-973	Senegalia mellifera	0.55	0.85	Acacia - not included
O-974	Senegalia nigrescens	0.74	0.92	Acacia - not included
O-975	Senegalia pennata	0.62	0.80	Acacia - not included
O-976	Senegalia pentagona	0.75	0.93	Acacia - not included
O-977	Senegalia persiciflora	0.80	0.93	Acacia - not included
O-978	Senegalia pervillei	0.81	0.94	Acacia - not included
O-979	Senegalia polyacantha	0.57	0.81	Acacia - not included
O-980	Senegalia rovumae	0.82	0.94	Acacia - not included
O-981	Senegalia sakalava	0.75	0.91	Acacia - not included
O-982	Senegalia schweinfurthii	0.69	0.90	Acacia - not included
O-983	Senegalia senegal	0.43	0.78	Acacia - not included
984	Setaria appendiculata	0.84	0.95	Panicoideae
985	Setaria atrata	0.88	0.94	Panicoideae
986	Setaria barbata	0.60	0.86	Panicoideae
987	Setaria finita	0.91	0.98	Panicoideae
988	Setaria homonyma	0.74	0.93	Panicoideae
989	Setaria humbertiana	0.94	0.97	Panicoideae
990	Setaria incrassata	0.68	0.87	Panicoideae
991	Setaria italica	0.54	0.80	Panicoideae
992	Setaria kagerensis	0.89	0.98	Panicoideae
993	Setaria lindenbergiana	0.87	0.96	Panicoideae
994	Setaria longiseta	0.75	0.92	Panicoideae
995	Setaria madecassa	0.78	0.91	Panicoideae
996	Setaria megaphylla	0.67	0.88	Panicoideae
997	Setaria nigrirostris	0.66	0.90	Panicoideae
998	Setaria obtusifolia	0.88	0.98	Panicoideae
999	Setaria orthosticha	0.79	0.92	Panicoideae
1000	Setaria parviflora	0.59	0.86	Panicoideae
1001	Setaria petiolata	0.88	0.96	Panicoideae
1002	Setaria poiretiana	0.85	0.98	Panicoideae
1003	Setaria pseudaristata	0.85	0.94	Panicoideae

	species name	TSS	AUC	group
1004	Setaria pumila	0.50	0.79	Panicoideae
1005	Setaria restioidea	0.83	0.95	Panicoideae
1006	Setaria rigida	0.88	0.98	Panicoideae
1007	Setaria sagittifolia	0.83	0.92	Panicoideae
1008	Setaria sphacelata	0.57	0.84	Panicoideae
1009	Setaria sulcata	0.82	0.95	Panicoideae
1010	Setaria vatkeana	0.84	0.94	Panicoideae
1011	Setaria verticillata	0.46	0.77	Panicoideae
1012	Setaria viridis	0.62	0.86	Panicoideae
1013	Setaria welwitschii	0.69	0.80	Panicoideae
1014	Sorghastrum incompletum	0.73	0.89	Panicoideae
1015	Sorghastrum nudipes	0.87	0.96	Panicoideae
1016	Sorghastrum stipoides	0.66	0.86	Panicoideae
1017	Sporobolus acinifolius	0.82	0.95	Chloridoideae
1018	Sporobolus africanus	0.64	0.86	Chloridoideae
1019	Sporobolus agrostoides	0.60	0.85	Chloridoideae
1020	Sporobolus albicans	0.90	0.98	Chloridoideae
1021	Sporobolus bechuanicus	0.96	0.99	Chloridoideae
1022	Sporobolus centrifugus	0.78	0.91	Chloridoideae
1023	Sporobolus confinis	0.66	0.86	Chloridoideae
1024	Sporobolus congoensis	0.76	0.91	Chloridoideae
1025	Sporobolus consimilis	0.57	0.82	Chloridoideae
1026	Sporobolus cordofanus	0.48	0.78	Chloridoideae
1027	Sporobolus coromandelianus	0.74	0.90	Chloridoideae
1028	Sporobolus dinklagei	0.86	0.93	Chloridoideae
1029	Sporobolus discosporus	0.88	0.96	Chloridoideae
1030	Sporobolus elatior	0.72	0.83	Chloridoideae
1031	Sporobolus engleri	0.68	0.89	Chloridoideae
1032	Sporobolus festivus	0.58	0.81	Chloridoideae
1033	Sporobolus fimbriatus	0.75	0.90	Chloridoideae
1034	Sporobolus geminatus	0.89	0.97	Chloridoideae
1035	Sporobolus halophilus	0.74	0.82	Chloridoideae
1036	Sporobolus helvolus	0.57	0.85	Chloridoideae
1037	Sporobolus indicus	0.67	0.89	Chloridoideae
1038	Sporobolus infirmus	0.79	0.92	Chloridoideae
1039	Sporobolus ioclados	0.60	0.87	Chloridoideae
1040	Sporobolus ludwigii	0.94	0.98	Chloridoideae
1041	Sporobolus macranthelus	0.65	0.85	Chloridoideae
1042	Sporobolus micranthus	0.82	0.92	Chloridoideae
1043	Sporobolus microprotus	0.71	0.89	Chloridoideae
1044	Sporobolus molleri	0.78	0.94	Chloridoideae
1045	Sporobolus myrianthus	0.79	0.94	Chloridoideae
1046	Sporobolus natalensis	0.78	0.93	Chloridoideae
1047	Sporobolus nebulosus	0.90	0.97	Chloridoideae
1048	Sporobolus nervosus	0.86	0.95	Chloridoideae
1049	Sporobolus niliacus	0.88	0.95	Chloridoideae
1050	Sporobolus nitens	0.88	0.98	Chloridoideae
1051	Sporobolus panicoides	0.81	0.94	Chloridoideae
1052	Sporobolus paniculatus	0.60	0.86	Chloridoideae
1053	Sporobolus pectinatus	0.91	0.97	Chloridoideae
1054	Sporobolus pectinellus	0.72	0.90	Chloridoideae
1055	Sporobolus pellucidus	0.68	0.85	Chloridoideae
1056	Sporobolus perrieri	0.77	0.91	Chloridoideae
1057	Sporobolus pilifer	0.82	0.92	Chloridoideae
1058	Sporobolus pyramidalis	0.63	0.87	Chloridoideae
1059	Sporobolus rigidifolius	0.88	0.93	Chloridoideae
1060	Sporobolus robustus	0.73	0.90	Chloridoideae
1061	Sporobolus ruspolianus	0.91	0.96	Chloridoideae
1062	Sporobolus salsus	0.92	0.96	Chloridoideae
1063	Sporobolus sanguineus	0.73	0.89	Chloridoideae
1064	Sporobolus spicatus	0.44	0.77	Chloridoideae
1065	Sporobolus stapfianus	0.79	0.92	Chloridoideae
1066	Sporobolus stolzii	0.72	0.85	Chloridoideae
1067	Sporobolus subglobosus	0.82	0.91	Chloridoideae
1068	Sporobolus subtilis	0.76	0.92	Chloridoideae
1069	Sporobolus subulatus	0.84	0.93	Chloridoideae
1070	Sporobolus tenellus	0.93	0.98	Chloridoideae
1071	Sporobolus tenuissimus	0.68	0.91	Chloridoideae
1072	Sporobolus virginicus	0.60	0.85	Chloridoideae
1073	Sporobolus welwitschii	0.87	0.96	Chloridoideae
1074	Stapfochloa lamproparia	0.88	0.95	Chloridoideae
1075	Steinchisma laxum	0.76	0.94	Panicoideae

	species name	TSS	AUC	group
1076	Stenotaphrum dimidiatum	0.74	0.91	Panicoideae
1077	Stenotaphrum oostachyum	0.74	0.87	Panicoideae
1078	Stenotaphrum secundatum	0.62	0.89	Panicoideae
1079	Stereochlaena cameronii	0.83	0.92	Panicoideae
1080	Stipagrostis amabilis	0.95	0.99	Aristidoideae
1081	Stipagrostis ciliata	0.68	0.89	Aristidoideae
1082	Stipagrostis giessii	0.82	0.95	Aristidoideae
1083	Stipagrostis hirtigluma	0.63	0.87	Aristidoideae
1084	Stipagrostis hochstetteriana	0.83	0.96	Aristidoideae
1085	Stipagrostis namaquensis	0.85	0.97	Aristidoideae
1086	Stipagrostis obtusa	0.80	0.96	Aristidoideae
1087	Stipagrostis subacaulis	0.90	0.96	Aristidoideae
1088	Stipagrostis uniplumis	0.54	0.81	Aristidoideae
1089	Tarigidia aequiglumis	0.78	0.96	Panicoideae
1090	Terminalia albida	0.79	0.92	Combretaceae
1091	Terminalia ankaranensis	0.87	0.93	Combretaceae
1092	Terminalia avicennioides	0.80	0.91	Combretaceae
1093	Terminalia boivinii	0.76	0.92	Combretaceae
1094	Terminalia brachystemma	0.82	0.94	Combretaceae
1095	Terminalia brownii	0.55	0.86	Combretaceae
1096	Terminalia calcicola	0.81	0.87	Combretaceae
1097	Terminalia calophylla	0.76	0.86	Combretaceae
1098	Terminalia cephalota	0.95	0.99	Combretaceae
1099	Terminalia crenata	0.94	0.98	Combretaceae
1100	Terminalia cyanocarpa	0.85	0.97	Combretaceae
1101	Terminalia disjuncta	0.98	0.98	Combretaceae
1102	Terminalia divaricata	0.88	0.97	Combretaceae
1103	Terminalia fatraea	0.92	0.96	Combretaceae
1104	Terminalia gazensis	0.96	0.98	Combretaceae
1105	Terminalia gracilipes	0.73	0.90	Combretaceae
1106	Terminalia griffithsiana	0.96	0.99	Combretaceae
1107	Terminalia kaiseriana	0.85	0.94	Combretaceae
1108	Terminalia kilimandscharica	0.71	0.93	Combretaceae
1109	Terminalia laxiflora	0.79	0.90	Combretaceae
1110	Terminalia leandriana	0.94	0.99	Combretaceae
1111	Terminalia macroptera	0.79	0.91	Combretaceae
1112	Terminalia mantaliopsis	0.60	0.82	Combretaceae
1113	Terminalia neotaliala	0.76	0.87	Combretaceae
1114	Terminalia ombrophila	0.89	0.93	Combretaceae
1115	Terminalia orbicularis	0.74	0.90	Combretaceae
1116	Terminalia perrieri	0.79	0.94	Combretaceae
1117	Terminalia phanerophlebia	0.86	0.97	Combretaceae
1118	Terminalia polycarpa	0.84	0.95	Combretaceae
1119	Terminalia prunioides	0.75	0.93	Combretaceae
1120	Terminalia randii	0.84	0.91	Combretaceae
1121	Terminalia rhopalophora	0.83	0.91	Combretaceae
1122	Terminalia septentrionalis	0.98	1.00	Combretaceae
1123	Terminalia sericea	0.78	0.91	Combretaceae
1124	Terminalia seyrigii	0.73	0.91	Combretaceae
1125	Terminalia spinosa	0.69	0.93	Combretaceae
1126	Terminalia stenostachya	0.77	0.94	Combretaceae
1127	Terminalia stuhlmannii	0.94	0.99	Combretaceae
1128	Terminalia subserrata	0.90	0.97	Combretaceae
1129	Terminalia sulcata	0.99	1.00	Combretaceae
1130	Terminalia trichopoda	0.91	0.95	Combretaceae
1131	Terminalia tricristata	0.87	0.96	Combretaceae
1132	Terminalia tropophylla	0.71	0.93	Combretaceae
1133	Terminalia ulexoides	0.72	0.92	Combretaceae
1134	Terminalia urschii	1.00	1.00	Combretaceae
1135	Tessmannia burttii	0.87	0.95	Detarioideae
1136	Tessmannia camoneana	0.92	0.96	Detarioideae
1137	Tessmannia densiflora	0.91	0.97	Detarioideae
1138	Tessmannia dewildemaniana	0.81	0.95	Detarioideae
1139	Tetrachaete elionuroides	0.66	0.88	Chloridoideae
1140	Tetrapogon bidentatus	0.75	0.92	Chloridoideae
1141	Tetrapogon cenchriformis	0.65	0.87	Chloridoideae
1142	Tetrapogon ferrugineus	0.95	0.96	Chloridoideae
1143	Tetrapogon tenellus	0.66	0.92	Chloridoideae
1144	Tetrapogon villosus	0.56	0.84	Chloridoideae
1145	Thelepogon elegans	0.76	0.92	Panicoideae
1146	Themeda quadrivalvis	0.60	0.83	Panicoideae
1147	Themeda triandra	0.57	0.84	Panicoideae

	species name	TSS	AUC	group
1148	Thuarea involuta	0.87	0.96	Panicoideae
1149	Thuarea perrieri	0.70	0.90	Panicoideae
1150	Trachypogon chevalieri	0.86	0.97	Panicoideae
1151	Trachypogon spicatus	0.66	0.89	Panicoideae
1152	Tricholaena capensis	0.89	0.98	Panicoideae
1153	Tricholaena monachne	0.70	0.87	Panicoideae
1154	Tricholaena teneriffae	0.67	0.89	Panicoideae
1155	Trichoneura ciliata	0.96	0.99	Chloridoideae
1156	Trichoneura eleusinoides	0.62	0.81	Chloridoideae
1157	Trichoneura grandiglumis	0.85	0.96	Chloridoideae
1158	Trichoneura mollis	0.73	0.88	Chloridoideae
1159	Trichoneura schlechteri	0.89	0.97	Chloridoideae
1160	Trichopteryx dregeana	0.79	0.94	Panicoideae
1161	Trichopteryx elegantula	0.86	0.95	Panicoideae
1162	Trichopteryx fruticulosa	0.86	0.96	Panicoideae
1163	Trichopteryx marungensis	0.78	0.94	Panicoideae
1164	Trichopteryx stolziana	0.88	0.94	Panicoideae
1165	Trigonochloa uniflora	0.82	0.94	Chloridoideae
1166	Tripogon curvatus	0.83	0.93	Chloridoideae
1167	Tripogon leptophyllus	0.70	0.83	Chloridoideae
1168	Tripogon major	0.75	0.91	Chloridoideae
1169	Tripogon subtilissimus	0.77	0.92	Chloridoideae
1170	Triraphis andropogonoides	0.90	0.98	Chloridoideae
1171	Triraphis pumilio	0.78	0.92	Chloridoideae
1172	Triraphis purpurea	0.85	0.95	Chloridoideae
1173	Triraphis ramosissima	0.87	0.97	Chloridoideae
1174	Triraphis schinzii	0.88	0.96	Chloridoideae
1175	Tristachya bequaertii	0.94	0.98	Panicoideae
1176	Tristachya biseriata	0.89	0.98	Panicoideae
1177	Tristachya hubbardiana	0.99	0.99	Panicoideae
1178	Tristachya humbertii	0.85	0.91	Panicoideae
1179	Tristachya leucothrix	0.83	0.96	Panicoideae
1180	Tristachya lualabaensis	0.98	0.98	Panicoideae
1181	Tristachya nodiglumis	0.80	0.95	Panicoideae
1182	Tristachya pedicellata	0.91	0.98	Panicoideae
1183	Tristachya rehmannii	0.88	0.98	Panicoideae
1184	Tristachya superba	0.79	0.93	Panicoideae
1185	Tristachya thollonii	0.77	0.90	Panicoideae
1186	Tristachya viridearistata	0.95	0.98	Panicoideae
1187	Urelytrum agropyroides	0.80	0.94	Panicoideae
1188	Urelytrum annuum	0.87	0.96	Panicoideae
1189	Urelytrum digitatum	0.90	0.96	Panicoideae
1190	Urelytrum giganteum	0.86	0.97	Panicoideae
1191	Urelytrum henrardii	0.95	0.97	Panicoideae
1192	Urelytrum muricatum	0.89	0.97	Panicoideae
1193	Urochloa arrecta	0.74	0.94	Panicoideae
1194	Urochloa brachyura	0.83	0.95	Panicoideae
1195	Urochloa brevispicata	0.85	0.93	Panicoideae
1196	Urochloa brizantha	0.67	0.89	Panicoideae
1197	Urochloa comata	0.71	0.92	Panicoideae
1198	Urochloa deflexa	0.52	0.81	Panicoideae
1199	Urochloa dictyoneura	0.72	0.92	Panicoideae
1200	Urochloa echinolaenoides	0.94	0.99	Panicoideae
1201	Urochloa eminii	0.69	0.91	Panicoideae
1202	Urochloa jubata	0.73	0.87	Panicoideae
1203	Urochloa leersioides	0.58	0.88	Panicoideae
1204	Urochloa mutica	0.61	0.88	Panicoideae
1205	Urochloa oligobrachiata	0.75	0.89	Panicoideae
1206	Urochloa oligotricha	0.79	0.92	Panicoideae
1207	Urochloa panicoides	0.60	0.85	Panicoideae
1208	Urochloa platyrrhachis	0.97	0.98	Panicoideae
1209	Urochloa ramosa	0.59	0.78	Panicoideae
1210	Urochloa reptans	0.68	0.87	Panicoideae
1211	Urochloa rudis	0.77	0.86	Panicoideae
1212	Urochloa sclerochlaena	0.88	0.97	Panicoideae
1213	Urochloa setigera	0.70	0.90	Panicoideae
1214	Urochloa trichopus	0.55	0.86	Panicoideae
1215	Urochloa villosa	0.51	0.81	Panicoideae
1216	Urochloa xantholeuca	0.56	0.82	Panicoideae
1217	Vachellia abyssinica	0.80	0.93	Acacia
1218	Vachellia arenaria	0.94	0.99	Acacia
1219	Vachellia bellula	0.94	0.99	Acacia

	species name	TSS	AUC	group
1220	Vachellia bussei	0.68	0.92	Acacia
1221	Vachellia davyi	0.81	0.95	Acacia
1222	Vachellia dolichocephala	0.73	0.90	Acacia
1223	Vachellia drepanolobium	0.69	0.92	Acacia
1224	Vachellia erioloba	0.76	0.91	Acacia
1225	Vachellia etbaica	0.63	0.89	Acacia
1226	Vachellia exuvialis	0.92	0.98	Acacia
1227	Vachellia farnesiana	0.51	0.79	Acacia
1228	Vachellia flava	0.64	0.84	Acacia
1229	Vachellia gerrardii	0.55	0.84	Acacia
1230	Vachellia grandicornuta	0.83	0.96	Acacia
1231	Vachellia haematoxylon	0.94	0.99	Acacia
1232	Vachellia hebeclada	0.76	0.94	Acacia
1233	Vachellia hockii	0.63	0.84	Acacia
1234	Vachellia horrida	0.75	0.93	Acacia
1235	Vachellia karroo	0.69	0.88	Acacia
1236	Vachellia kirkii	0.57	0.87	Acacia
1237	Vachellia lahai	0.82	0.94	Acacia
1238	Vachellia luederitzii	0.77	0.93	Acacia
1239	Vachellia nebrownii	0.83	0.95	Acacia
O-1240	Vachellia nilotica	0.37	0.74	Acacia
1241	Vachellia rehmanniana	0.71	0.90	Acacia
1242	Vachellia robusta	0.55	0.85	Acacia
O-1243	Vachellia seyal	0.43	0.74	Acacia
1244	Vachellia sieberiana	0.53	0.78	Acacia
1245	Vachellia stuhlmannii	0.70	0.90	Acacia
1246	Vachellia swazica	0.89	0.96	Acacia
O-1247	Vachellia tortilis	0.36	0.74	Acacia
1248	Vachellia tortuosa	0.61	0.87	Acacia
1249	Vachellia xanthophloea	0.73	0.91	Acacia
1250	Vachellia zanzibarica	0.77	0.93	Acacia
1251	Yvesia madagascariensis	0.71	0.86	Panicoideae
1252	Zenkerella capparidacea	0.96	0.99	Detarioideae
1253	Zenkerella egregia	0.84	0.95	Detarioideae
1254	Zonotriche decora	0.98	0.99	Panicoideae
1255	Zonotriche inamoena	0.90	0.97	Panicoideae

Table S2: Comparison of models using our lineage-defined classes and growth-form-defined classes from [2] using WAIC: Widely Applicable Information Criterion. pWAIC is the effective number of parameters, SE is the standard error of the WAIC estimate. Delta is the difference between the lineage (-L) and the growth form (-GF) models, for NPP, Burnt area, Coefficient of variation of burnt area and Cattle density models. A Delta larger than four standard errors is considered as decisive evidence for better model performance.

Model	WAIC	pWAIC	SE	Delta
NPP-L	-650.60	8.21	106.49	
NPP-GF	865.37	16.01	102.68	1515.97
Burnt area-L	24305.48	7.40	65.45	
Burnt area-GF	28626.48	8.74	69.70	4320.99
CV burnt area-L	5774.88	11.24	145.69	
CV burnt area-GF	6926.39	17.42	155.64	1151.51
Cattle density-L	24613.35	141.26	987.52	
Cattle density-GF	30272.58	144.51	1018.65	5659.23

Table S3: Comparison of models using our lineage-defined classes and growth-form-defined classes from [2] using LOO: efficient approximate leave-one-out (LOO) cross-validation for Bayesian models using Pareto smoothed importance sampling. elpd_loo is the expected log pointwise predictive density, p_loo the effective number of parameters, SE is the standard error of the elpd_loo estimate. Delta is the difference between the lineage (-L) and the growth form (-GF) models, for NPP, Burnt area, Coefficient of variation of burnt area and Cattle density models. A Delta larger than four standard errors is considered as decisive evidence for better model performance.

Model	elpd_loo	p_loo	SE	Delta
NPP-L	325.28	8.23	53.25	
NPP-GF	-432.83	16.16	51.35	758.11
Burnt area-L	-12152.75	7.41	32.72	
Burnt area-GF	-14313.26	8.76	34.85	2160.51
CV burnt area-L	-2887.46	11.25	72.85	
CV burnt area-GF	-3463.34	17.57	77.82	575.89
Cattle density-L	-12255.94	90.52	446.93	
Cattle density-GF	-15093.93	102.16	476.30	2838.00

Table S4: Comparison of models using our lineage-defined classes, growth-form-defined classes from [2] and floristically-defined classes from [1] using Gelman's R2 for Bayesian linear models [5] to assess absolute goodness-of-fit. Shown is the R2 for the lineage (-L), the growth form (-GF) and floristic map (-WH) models, for NPP, Burnt area, Coefficient of variation of burnt area and Cattle density models. Please note that the number of classes and thus flexibility differs between models, with 7 for the lineage, 8 for the growth-form and 26 for the White floristic map model which is not inherently penalized by the R2 statistic.

Model	Gelman's R2
NPP-L	0.68
NPP-GF	0.51
NPP-WH	0.64
Burnt area-L	0.27
Burnt area-GF	0.19
Burnt area-WH	0.35
CV burnt area-L	0.34
CV burnt area-GF	0.30
CV burnt area-WH	0.38
Cattle density-L	0.10
Cattle density-GF	0.08
Cattle density-WH	0.08

Table S5: Comparison of models using our lineage-defined classes and floristically defined classes from [1] using WAIC: Widely Applicable Information Criterion. pWAIC is the effective number of parameters, SE is the standard error of the WAIC estimate. Delta is the difference between the lineage (-L) and the White map (-WH) models, for NPP, Burnt area, Coefficient of variation of burnt area and Cattle density models. A Delta larger than four standard errors is considered as decisive evidence for better model performance.

Model	WAIC	pWAIC	SE	Delta
NPP-L	-650.60	8.21	106.49	
NPP-WH	-73.93	21.19	139.40	576.67
Burnt area-L	24305.48	7.40	65.45	
Burnt area-WH	20835.03	17.39	67.37	-3470.46
CV burnt area-L	5774.88	11.24	145.69	
CV burnt area-WH	4967.71	21.28	124.71	-807.17
Cattle density-L	24613.35	141.26	987.52	
Cattle density-WH	22830.11	131.11	905.46	-1783.24

Table S6: Comparison of models using our lineage-defined classes and floristically defined classes from [1] using LOO: efficient approximate leave-one-out (LOO) cross-validation for Bayesian models using Pareto smoothed importance sampling. elpd_loo is the expected log pointwise predictive density, p_loo the effective number of parameters, SE is the standard error of the elpd_loo estimate. Delta is the difference between the lineage (-L) and the White map (-WH) models, for NPP, Burnt area, Coefficient of variation of burnt area and Cattle density models. A Delta larger than four standard errors is considered as decisive evidence for better model performance.

Model	elpd_loo	p_loo	SE	Delta
NPP-L	325.28	8.23	53.25	
NPP-WH	36.52	21.64	69.70	288.77
Burnt area-L	-12152.75	7.41	32.72	
Burnt area-WH	-10417.75	17.63	33.69	-1735.00
CV burnt area-L	-2887.46	11.25	72.85	
CV burnt area-WH	-2484.00	21.43	62.35	-403.45
Cattle density-L	-12255.94	90.52	446.93	
Cattle density-WH	-11373.24	89.29	421.02	-882.70

Table S7: Detailed acknowledgement of species data sources. For plot data, absence points were derived if a survey was listed as a full species inventory in the respective dataset.

	data type used	source
1	presence records	RAINBIO database. Dauby, G., Zaiss, R., Blach-Overgaard, A., et al. (2016)<br>reposited in BIEN V4 https://bien.nceas.ucsb.edu/bien/
2	presence records	GBIF occurrence download. Please refer to below links for specified data contributors:<br>https://doi.org/10.15468/dl.7ncqkp (Combretaceae)<br>https://doi.org/10.15468/dl.aj42xd (Commiphora)<br>https://doi.org/10.15468/dl.dbpzpz (Panicoideae)<br>https://doi.org/10.15468/dl.sskxdc (Chloridoideae)<br>https://doi.org/10.15468/dl.e6bmjk (Aristidoideae)<br>https://doi.org/10.15468/dl.n22bsu (Acacia s.l.)<br>https://doi.org/10.15468/dl.2pc5p6 (Detarioideae)
3	plot - presence and absence	afrotroptree dataset.<br>associated publication: Aleman, J.C., Fayolle, A., Favier, C., et al. (2020)
4	plot - presence and absence	FLOTROP dataset.<br>reposited on GBIF: https://doi.org/10.15468/dl.52g6un
5	plot - presence and absence	ACKDAT dataset.<br>associated publication: Rutherford, M., Powrie, L. and Midgley, G. (2003)
6	plot - presence and absence	National Vegetation Database.<br>associated publication: Rutherford, M., Mucina, L. and Powrie, L. (2012)
7	plot - presence and absence	West Africa dataset.<br>reposited on GBIF: https://doi.org/10.15468/dl.28nngh

Table S8: Summary of how categories on the White map were merged to amount to the same number of categories as on the phytoclime map for formal comparison. Overlap shows the fraction of pixels in which a given White map category coincides with a phytoclime map category, as opposed to the other six categories. The categories "0/not savanna" were preemptively excluded (therefore automatically an overlap of 1).

phytoclime equivalent	White map category (mapping unit)	overlap
0	not savanna (1-21,23-24,33-34,38-39,49-80)	1
1	dry deciduous forest-grassland mosaic (22a)	0.50
1	dry Miombo (26)	0.43
1	undifferentiated woodland (North Zambezian)(29c)	0.43
1	undifferentiated woodland (South Zambezian)(29d)	0.62
1	deciduous thicket (Itigi) (40)	0.46
1	deciduous thicket (malagasy) (41)	0.70
1	evergreen bushland-Acacia parkland (45)	0.87
1	bushland & thicket mosaics (malagasy) (46)	0.94
1	Tugela basin bushland (48)	1
2	Mopane woodland and scrub woodland(28)	0.53
2	undifferentiated woodland-Acacia bushland (Zambezian) (35a)	0.97
2	undifferentiated woodland-Acacia bushland (Windhoek Mountains) (35c)	1
2	Mopane-Namib transition (36)	0.50
2	Kalahari Acacia parkland-bushland (44)	0.95
3	Acacia-Commiphora bushland (42)	0.45
3	Sahel Acacia parkland-bushland (43)	0.78
4	Sudanian Isoberlinia woodland (27,30)	0.78
4	undifferentiated woodland (Ethiopian) (29b)	0.33
5	undifferentiated woodland (Sudanian)(29a)	0.69
5	undifferentiated woodland-Acacia bushland (Ethiopian)(35b)	0.54
6	deciduous forest-grassland mosaic (malagasy) (22b)	0.61
7	wet Miombo (25)	0.76
7	mosaic woodlands (Zambezian) (31)	0.39
7	mosaic woodlands (Jos plateau) (32)	0.47
7	secondary Acacia parkland (37)	0.36
7	bushland & thicket mosaics (*Brachystegia bakerana*) (47)	0.77

Table S9: Summary of how categories on the White map were merged to amount to the same number of categories as on the growth-form defined map [2] for formal comparison in Fig. S2. Overlap shows the fraction of pixels in which a given growth-form map category coincides with a phytoclime map category, as opposed to the other six categories. Category numbers are slightly altered from [2] as we cropped out the non-savanna region as category 0, "not savanna". For easier comparability, the row order is the same as in Tab. S8

functional class equivalent	White map category (mapping unit)	overlap
0	not savanna (1-21,23-24,33-34,38-39,49-80)	1
1	dry deciduous forest-grassland mosaic (22a)	0.11
12	dry Miombo (26)	0.41
3	undifferentiated woodland (North Zambezian)(29c)	0.13
3	undifferentiated woodland (South Zambezian)(29d)	0.28
2	deciduous thicket (Itigi) (40)	0.006
10	deciduous thicket (malagasy) (41)	0.008
4	evergreen bushland-Acacia parkland (45)	0.10
2	bushland & thicket mosaics (malagasy) (46)	0.01
3	Tugela basin bushland (48)	0.02
1	Mopane woodland and scrub woodland(28)	0.20
1	undifferentiated woodland-Acacia bushland (Zambezian) (35a)	0.23
7	undifferentiated woodland-Acacia bushland (Windhoek Mountains) (35c)	0.1
7	Mopane-Namib transition (36)	0.08
5	Kalahari Acacia parkland-bushland (44)	0.67
6	Acacia-Commiphora bushland (42)	0.26
8	Sahel Acacia parkland-bushland (43)	0.56
10	Sudanian Isoberlinia woodland (27,30)	0.41
11	undifferentiated woodland (Ethiopian) (29b)	0.03
6	undifferentiated woodland (Sudanian)(29a)	0.45
10	undifferentiated woodland-Acacia bushland (Ethiopian)(35b)	0.03
10	deciduous forest-grassland mosaic (malagasy) (22b)	0.06
14	wet Miombo (25)	0.93
12	mosaic woodlands (Zambezian) (31)	0.20
11	mosaic woodlands (Jos plateau) (32)	0.04
12	secondary Acacia parkland (37)	0.04
9	bushland & thicket mosaics (*Brachystegia bakerana*) (47)	0.24

References

[1] F. White. *The vegetation of Africa: a descriptive memoir to accompany the UNESCO/AETFAT/UNSO vegetation map of Africa.* United Nations, Paris, France, 1983.

[2] T. Conradi, S. I. Higgins, G. F. Midgley, H. Nottebrock, A. H. Schweiger, and J. A. Slingsby. An operational definition of the biome for global change research. *New Phytologist*, 227:1294–1306, 2020.

[3] R. A. Monserud and R. Leemans. Comparing global vegetation maps. *Ecological Modelling*, 62:275–293, 1992.

[4] L. Scrucca, C. Fraley, T.B. Murphy, and A.E. Raftery. *Model-Based Clustering, Classification, and Density Estimation Using mclust in R.* Chapman and Hall/CRC, 2023.

[5] A. Gelman, B. Goodrich, J. Gabry, and A. Vehtari. R-Squared for Bayesian Regression Models. *The American Statistician*, 73:307–309, 2019.
